# Double-shelled hollow rods assembled from nitrogen/sulfur-codoped carbon coated indium oxide nanoparticles as excellent photocatalysts

**DOI:** 10.1038/s41467-019-10302-0

**Published:** 2019-05-22

**Authors:** Liming Sun, Rong Li, Wenwen Zhan, Yusheng Yuan, Xiaojun Wang, Xiguang Han, Yanli Zhao

**Affiliations:** 1 Jiangsu Key Laboratory of Green Synthetic Chemistry for Functional Materials, Department of Chemistry, School of Chemistry and Materials Science, Jiangsu Normal University, Xuzhou, 221116 P. R. China; 2 Division of Chemistry and Biological Chemistry, School of Physical and Mathematical Sciences, Nanyang Technological University, 21 Nanyang Link, Singapore, 637371 Singapore

**Keywords:** Photocatalysis, Metal-organic frameworks, Photocatalysis

## Abstract

Excellent catalytic activity, high stability and easy recovery are three key elements for fabricating efficient photocatalysts, while developing a simple method to fabricate such photocatalysts with these three features at the same time is highly challenging. In this study, we successfully synthesized double-shelled hollow rods (DHR) assembled by nitrogen (N) and sulfur (S)-codoped carbon coated indium(III) oxide (In_2_O_3_) ultra-small nanoparticles (N,S-C/In_2_O_3_ DHR). N,S-C/In_2_O_3_ DHR exhibits remarkable photocatalytic activity, high stability and easy recovery for oxidative hydroxylation reaction of arylboronic acid substrates. The catalyst recovery and surface area were well balanced through improved light harvesting, contributed by concurrently enhancing the reflection on the outer porous shell and the diffraction in the inside double-shelled hollow structure, and increased separation rate of photogenerated carriers. Photocatalytic mechanism was investigated to identify the main reactive species in the catalytic reactions. The electron separation and transfer pathway via N,S-codoped graphite/In_2_O_3_ interface was revealed by theoretical calculations.

## Abstract (web summary)

While photoredox catalysis presents exciting avenues for molecular transformations, balancing optimal photochemical and materials properties can be challenging. Here, the authors prepare carbon-coated In_2_O_3_ nanoparticles as recoverable photocatalysts for arylboronic acid oxidative hydroxylation.

## Introduction

Photoredox catalysis is a highly promising route to solar-to-chemical energy conversion in the form of organic synthesis under relatively mild conditions^1–9^. Among numerous photocatalysts studied in recent decades, indium oxide (In_2_O_3_) with narrow bandgap (∼2.8 eV), high optical transparency, excellent stability, and suitable band potentials (including valence band (VB) and conduction band (CB)) to induce redox reactions has attracted much attention as a visible-light-induced photocatalyst^10–15^. Compared with bulk In_2_O_3_, granular In_2_O_3_ nanoparticles have large surface areas, so they contain more active sites and display improved photocatalytic activity^16–18^. However, In_2_O_3_ nanoparticles still possess two undesirable disadvantages, i.e., the fast recombination of photogenerated carriers^19^ and the hard recovery in solution, which suppress their practical applications in photocatalytic field. Therefore, a rational design of nanosized granular In_2_O_3_ with high reactivity and easy recovery is highly desirable for extending their applications.

One effective approach to achieve the above-mentioned target is to assemble carbon-coated In_2_O_3_ nanoparticles into the superstructure with regular morphology and large size. On account of excellent mobility of charge carriers, the carbon layer can act as photogenerated electron acceptor to improve the separation efficiency of photogenerated carriers^20–22^. In particular, N,S-codoping sites with induced structure defects in carbon framework could increase the electron delocalization, further enhancing the separation efficiency of electrons and holes^23–25^. Thus, integrating N,S-codoped carbon layer with In_2_O_3_ nanoparticles would effectively enhance the photocatalytic activity. Moreover, assembling the resulted N,S-codoped carbon-coated In_2_O_3_ nanoparticles into the superstructure may facilitate the recovery of photocatalysts. However, there are two bottlenecks in this approach for fabricating efficient In_2_O_3_ photocatalysts. Firstly, it is difficult to control the coating of a carbon layer and introduce double heteroatoms into the carbon layer. In general, sufficient and intimate contact interface is the key factor to ensure efficient transfer of photogenerated carriers. However, most of the reported carbon materials as the substrate loaded on metal oxide nanoparticles had insufficient carbon/nanoparticle interface^26^. Uniformly coating a carbon layer on metal oxide nanoparticles is still a challenging task. In addition, conventional approaches for introducing heteroatoms into carbon materials have some unavoidable disadvantages, such as toxic precursors, special and/or sophisticated instruments, harsh conditions, and uneven distribution of doped atoms^27–30^. Realizing the co-doping of two types of heteroatoms is even harder. Therefore, it is highly needed to overcome the challenge toward fabricating ultrafine metal oxide nanoparticles coated by co-doped carbon layers. Secondly, it is difficult to balance the ease of photocatalyst recovery with the surface area. Although assembling N,S-codoped carbon-coated In_2_O_3_ ultrafine nanoparticles into photocatalysts with regular morphology and large size is a straightforward method to solve the problem of difficult recycling^31,32^, this solution would result in the decreases of surface areas, reactive sites and photocatalytic activity. Therefore, it is necessary to balance the effects of photocatalyst recovery and surface area by improving other factors affecting photocatalytic activity. To address these issues, we developed a promising approach to fabricate efficient photocatalysts assembled from N,S-codoped carbon uniformly coated In_2_O_3_ nanoparticles, aiming to achieve excellent catalytic activity and easy recovery at the same time.

Herein, double-shelled hollow rods (DHRs) assembled by N,S-codoped carbon uniformly coated In_2_O_3_ ultrafine nanoparticles (N,S-C/In_2_O_3_ DHR) were synthesized using a porous metal organic framework (MOF), i.e., MIL-68-In, as the template and 1,2-benzisothiazolin-3-one (BIT) as the modulator in one step. Since the carbon atom disperses homogeneously in the MOF structure at a molecular level^33–42^, the carbon layer in N,S-C/In_2_O_3_ DHR is uniformly distributed on the In_2_O_3_ surface to produce intimately contacted core-shell structure and maximize the interaction between the components, facilitating the separation of charge carriers. The obtained N,S-C/In_2_O_3_ DHR could be recycled from the solution through simple centrifugation. Moreover, N,S-C/In_2_O_3_ DHR performs remarkably in catalyzing a series of arylboronic acids under light irradiation. The photocatalytic time of N,S-C/In_2_O_3_ DHR required to achieve 99% yield was only half of the time needed by recently reported N-doped carbon-coated dodecahedral In_2_O_3_ (N-C/In_2_O_3_ HD)^43^. In addition, the as-prepared N,S-C/In_2_O_3_ DHR exhibits high photocatalytic activity under green (3 W LED lamp, 525 nm) and yellow (3 W LED lamp, 590 nm) lights, indicating that it broadens the response range of light to wider wavelength. This observation is of great significance for expanding the practical application of In_2_O_3_-based materials. However, N-C/In_2_O_3_ HD did not show any photocatalytic activity under green and yellow lights^43^. These comparison results suggest the importance of N,S-codoping for improving the photocatalytic activity of carbon-coated photocatalysts. Benefiting from the simultaneously increased reflection on the outer shell and diffraction on the hollow cavity of the N,S-codoped double-shelled hollow structure, and the enhanced separation rate of photogenerated carriers, the photocatalyst recovery and surface area were well balanced. The mechanism for the enhanced separation efficiency of photogenerated carriers by N,S codoping was clarified by theoretical calculations, i.e., the hybridization of N 2p, S 3p and C 2p increases the strength of interstitial states to further improve the transfer and separation efficiency of photogenerated carriers. We also investigated photocatalytic mechanism of the oxidative hydroxylation and confirmed the key active radical of this system in the photocatalysis. Finally, the microscopic pathway for the separation and migration of carriers in N,S-C/In_2_O_3_ DHR was revealed by theoretical calculations.

## Results

### Structure and composition of the photocatalysts

**Fig. 1:**
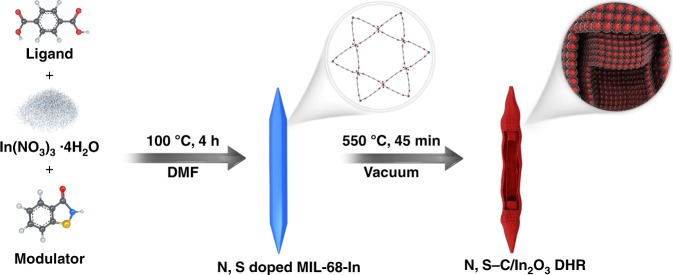
Schematic illustration showing the synthetic process of monodispersed N,S-C/In_2_O_3_ DHR

**Fig. 2:**
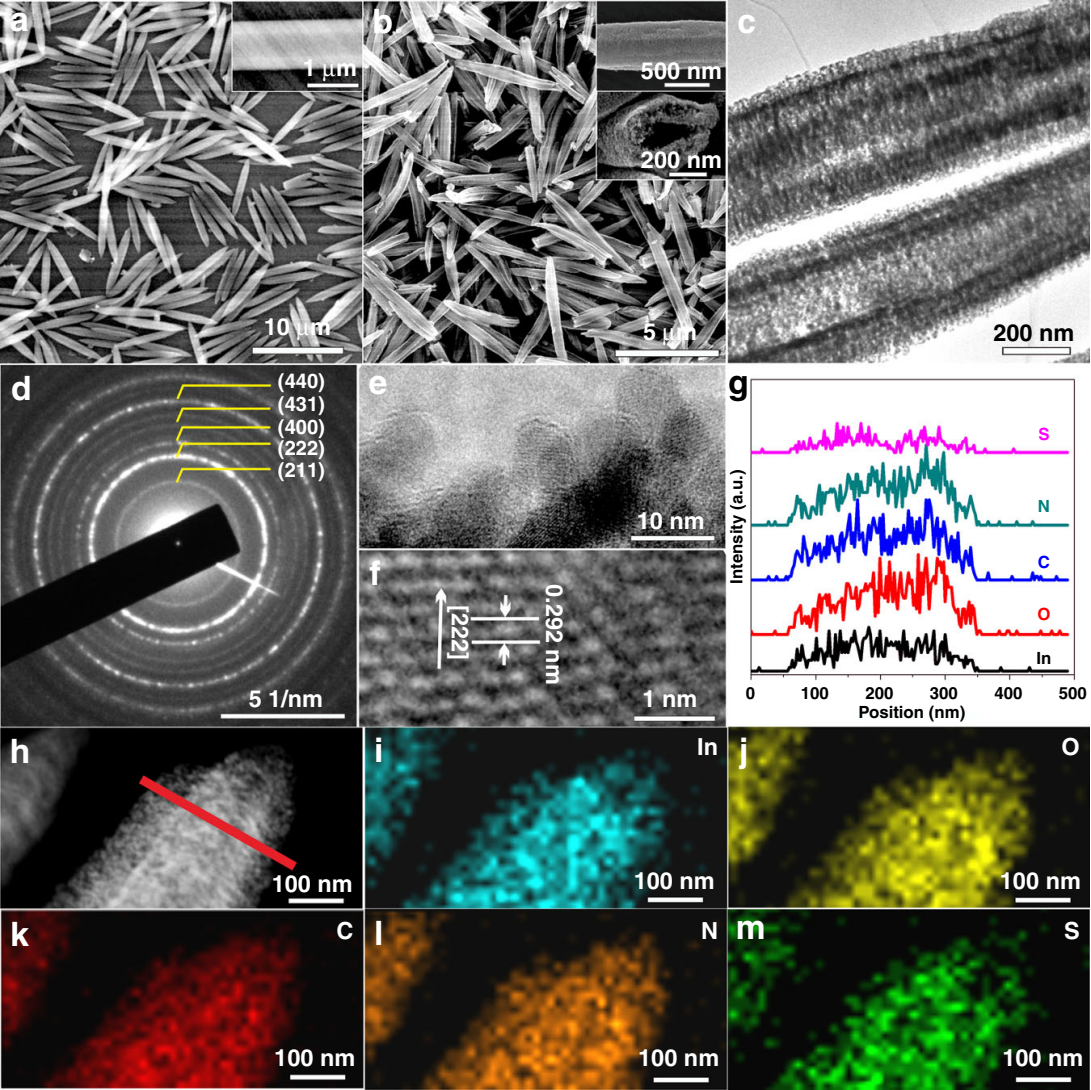
Morphology and structural characterizations. **a** SEM image of N,S-codoped MIL-68-In. **b** SEM images, **c** TEM image, **d** Corresponding SAED pattern, **e** Local enlarged TEM image, **f** HRTEM image, **g** Elemental profiles, **h** STEM image, and **i**–**m** EDX elemental mapping of In, O, C, N, and S for N,S-C/In_2_O_3_ DHR. Source data are provided as a Source Data file

The preparation of monodispersed double-shelled hollow rods (N,S-C/In_2_O_3_ DHR) assembled by N,S-codoped carbon-coated In_2_O_3_ ultrafine nanoparticles is schematically shown in Fig. 1. The experimental details are presented in the Supporting Information. During the synthetic process, BIT acted a modulator to control the growth of MIL-68-In crystals from indium metal ion and *p*-phthalic acid ligand, resulting in the formation of N,S-codoped MIL-68-In with the rod morphology. The length-width ratio of N,S-codoped MIL-In-68 rods could be controlled by adding different amounts of BIT^44,45^. Upon increasing the amount of BIT, the length-width ratio of N,S-codoped MIL-In-68 rods gradually decreases (Supplementary Fig. 1). A subsequent high-temperature annealing treatment of N,S-codoped MIL-68-In with the largest length-width ratio in vacuum atmosphere leads to the formation of N,S-C/In_2_O_3_ DHR. Typical diffraction peaks in the powder X-ray diffraction (XRD) pattern (Supplementary Fig. 2a) of N,S-codoped MIL-68-In can be well indexed to phase-pure MIL-68-In, which are in good agreement with simulated ones. Scanning electron microscopy (SEM) images of N,S-codoped MIL-68-In at different magnifications are shown in Fig. 2a. The size of N,S-codoped MIL-68-In crystals is 0.4–1.2 µm in width and 5–9 µm in length (Supplementary Fig. 3). Moreover, these N,S-codoped MIL-68-In crystals are highly monodispersed, showing well-defined rod morphology with smooth surface.

Notably, the BIT molecule provides copious N and S elements into MIL-68-In for enabling the creation of N and S codoped materials. To check the existence of N and S elements in MIL-68-In, the energy-dispersive X-ray (EDX) mapping of N,S-codoped MIL-68-In was measured to investigate the element distributions. The corresponding EDX maps of In, O, C, N, and S elements (Supplementary Fig. 4) indicate that all these elements are well dispersed in MIL-68-In. The thermal stability of N,S-codoped MIL-68-In is investigated by thermogravimetric analysis (TGA, Supplementary Fig. 5). According to the TGA curve, initial 6.7% weight lost between 400 and 500 ^o^C can be ascribed to the adsorbed and coordinated solvent molecules, and the steep weight loss (about 55.5%) is due to the carbonization of N,S-codoped MIL-68-In. Hence, annealing N,S-codoped MIL-68-In at 550 ^o^C in a vacuum atmosphere is sufficient to ensure complete conversion from MIL-68-In to In_2_O_3_.

Crystalline structure of the annealed product (N,S-C/In_2_O_3_ DHR) was explored by powder XRD (Supplementary Fig. 2b). All of the diffraction patterns correspond well with the cubic In_2_O_3_ (JCPDS card number 00–006–0416), indicating that the N,S-codoped MIL-68-In precursor is transformed to cubic In_2_O_3_ during the annealing treatment. The SEM image (Fig. 2b) indicates that the overall rod morphology of MIL-68-In is well preserved with 0.5–0.8 µm in width and 5–8 µm in length (Supplementary Fig. 6). The size of the annealed product is slightly smaller than that of N,S-codoped MIL-68-In, which is mainly attributed to partial collapse of MOF skeleton and the carbonization of organic ligand and modulator during the transformation process. The magnified observation (insert of Fig. 2b) reveals the micro-rod morphology with a double-shelled hollow structure, resulted from heterogeneous decomposition of the MIL-68-In MOF during the annealing process. The exterior MOF materials firstly decompose to form an In_2_O_3_ outer shell, and then the inner MOF structure is gradually carbonized under prolonged pyrolysis on account of large temperature gradient from the surface to the inner structure.

More structural details of double-shelled micro-rods are revealed by transmission electron microscopy (TEM). The low magnification TEM image (Fig. 2c) shows obviously brighter contrast, which demonstrates the double-shelled feature and porous nature of the resulting N,S-C/In_2_O_3_ DHR, corresponding well with the SEM observations. The well-defined rings of electron diffraction pattern (Fig. 2d) confirm the polycrystallinity of In_2_O_3_ rods. A high-magnification TEM survey (Fig. 2e) from a typical sample area shows that the porous rod structure of N,S-C/In_2_O_3_ DHR is really constituted by individual nanoparticles with the diameter about 10 nm. The TEM image also clearly exhibits a continuous carbon layer covered on the entire surface of nanoparticles. The surface carbon layer can increase the electrical conductivity of In_2_O_3_ nanoparticles, and thus improves the separation efficiency of photogenerated carriers. In addition, the external carbon layer can block the aggregation and increase the stability of the nanoparticles. Clear lattice fringes were observed from the high-resolution TEM (HRTEM) image (Fig. 2f), where 0.292 nm is assigned to the (222) interplane spacing of cubic In_2_O_3_, further confirming the cubic In_2_O_3_ phase of the nanoparticles. The presence of the In, C, O, S, and N elements in N,S-C/In_2_O_3_ DHR was proven by elemental distribution analysis, including the elemental line profiles (Fig. 2g) and elemental mapping. Scanning transmission electron microscopy (STEM) image indicates that the double-shelled structure of the In_2_O_3_ rods is indeed built by small nanoparticles (Fig. 2h). The elemental mapping results (Fig. 2i–m) demonstrate the uniform distribution of the In, C, O, S, and N within the N,S-C/In_2_O_3_ DHR structure.

During the pyrolysis, the indium ion in the MIL-68-In micro-rods is converted to In_2_O_3_ nanoparticles, and the external N,S-codoped carbon layer is formed by in situ carbonization. Therefore, the final annealed product is N,S-codoped carbon-coated In_2_O_3_ ultra-small nanoparticles with DHR morphology. The porous feature of N,S-C/In_2_O_3_ DHR was investigated by N_2_ adsorption/desorption isotherms (Supplementary Fig. 7). The Brunauer-Emmett-Teller (BET) measurements give a specific surface area of 37.6 m^2^/g, and the Barrett-Joyner-Halenda (BJH) pore size distribution is mainly centered at about 10–50 nm (insert of Supplementary Fig. 7). These results confirm that the N,S-C/In_2_O_3_ DHR obtained by the calcination of N,S-codoped MIL-68-In in vacuum atmosphere has DHR morphology with a mesoporous structure.

**Fig. 3:**
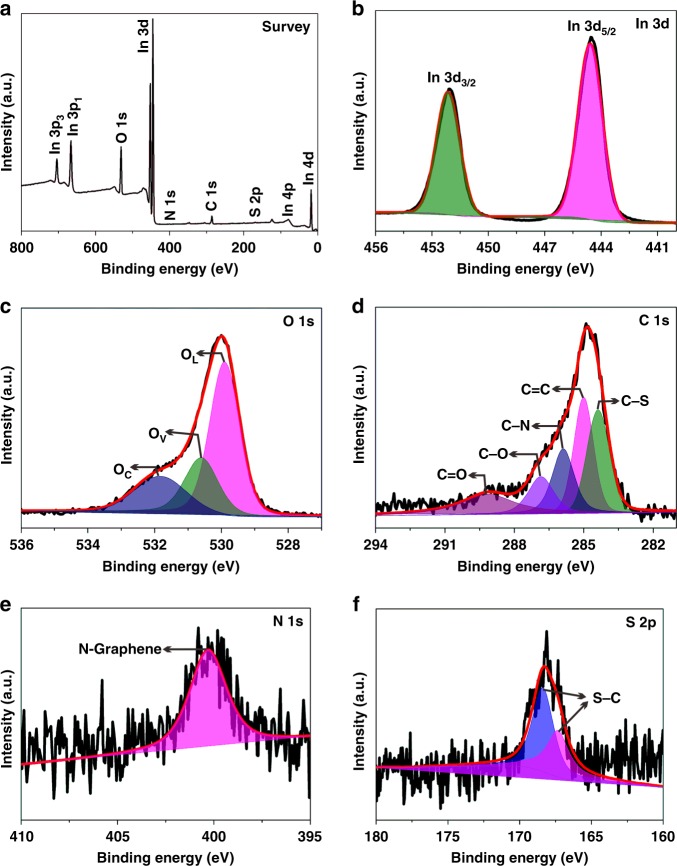
XPS spectra of N,S-C/In_2_O_3_ DHR. **a** Survey spectrum, and high-resolution spectra of **b** In 3d, **c** O 1s, **d** C 1s, **e** N 1s, and **f** S 2p. Source data are provided as a Source Data file

Chemical states of elements in the N,S-C/In_2_O_3_ DHR were measured by X-ray photoelectron spectroscopy (XPS). The survey spectrum (Fig. 3a) indicates the existence of In, O, N, S, and C elements in N,S-C/In_2_O_3_ DHR. The high-resolution XPS spectrum of In 3d shows two peaks corresponding to In 3d_3/2_ and In 3d_5/2_ (Fig. 3b). Higher binding energy at 444.5 eV corresponds to In 3d_5/2_ of In_2_O_3_, and the peak at 452.1 eV is in agreement with In 3d_3/2_ of In_2_O_3_. The high-resolution spectrum of O 1s displays an asymmetric curve (Fig. 3c), which consists of three peaks corresponding to three kinds of oxygen in N,S-C/In_2_O_3_ DHR. The three peaks at 529.8 eV (O_L_), 530.6 eV (O_V_) and 531.8 eV (O_C_) are assigned to lattice oxygen, oxygen-deficient region, and chemisorbed oxygen species (e.g., hydroxyl species), respectively^31,46^. The C 1s high-resolution spectrum (Fig. 3d) can be coherently fitted by five major peaks, assigning to C=O bonds (288.9 eV), C–O bonds (286.8 eV), C–N bonds (285.8 eV), C=C bonds (284.9 eV) and C–S bonds (284.2 eV). The existence of nitrogen elements in the N, S-C/In_2_O_3_ DHR structure is mainly ascribed to the N-graphene (Fig. 3e), which is also supported by C 1s spectrum. The S 2p spectrum (Fig. 3f) can be deconvoluted into two peaks, assigning to S–C (167.3 eV and 168.4 eV). These results further confirm that the N, S-C/In_2_O_3_ DHR is comprised of In_2_O_3_ coated by the N,S co-doped carbon layer. The N and S co-doping could improve the electronic transport efficiency and chemical activity of the carbon layer, which could in turn enhance the separation efficiency of carriers in photocatalytic reactions.

### Photophysical properties of the photocatalysts

**Fig. 4:**
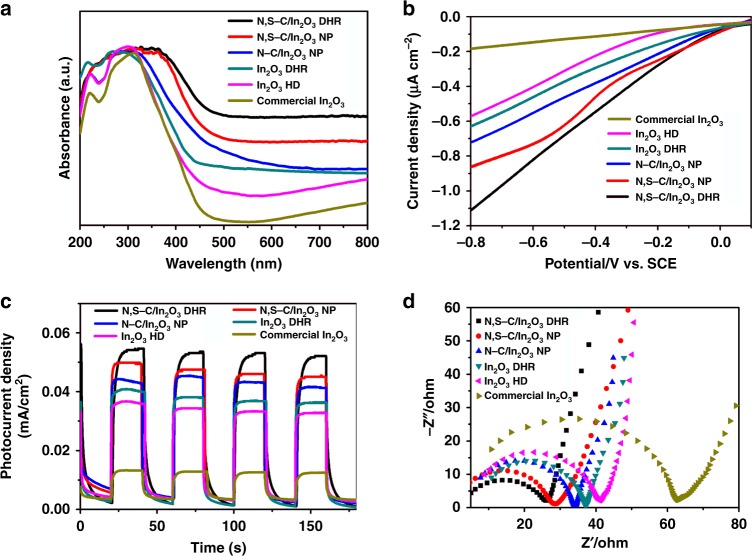
Optical absorption and photoelectric characterizations. **a** UV–vis absorption spectra, **b** LSV curves, **c** Photocurrent density measured at 0.2 V versus Hg/Hg_2_Cl_2_ under non-illuminated (i.e., in the dark) and illuminated conditions, and **d** EIS Nyquist plots for N,S-codoped In_2_O_3_ DHR, N,S-C/In_2_O_3_ NP, N-C/In_2_O_3_ NP, In_2_O_3_ DHR, In_2_O_3_ HD, and commercial In_2_O_3_. Source data are provided as a Source Data file

The first step of photocatalysis is the generation of electron-hole pairs under light irradiation. Thus, the UV–vis absorption spectrum of the obtained N,S-C/In_2_O_3_ DHR was measured and compared with that of N,S-C/In_2_O_3_ NP (In_2_O_3_ nanoparticles coated by N,S-codoped carbon, Supplementary Fig. 8), N-C/In_2_O_3_ NP (In_2_O_3_ nanoparticles coated by N-doped carbon, Supplementary Fig. 9), In_2_O_3_ DHR (one-dimensional DHRs assembled by In_2_O_3_ nanoparticles without the carbon layer, Supplementary Fig. 10), In_2_O_3_ HD (three-dimensional double-shelled hollow dodecahedron assembled by In_2_O_3_ nanoparticles without the carbon layer, Supplementary Figure 11), and commercial In_2_O_3_ in order to investigate the influence of unique structural features of N,S-C/In_2_O_3_ DHR on the light absorption ability. Figure 4a shows that the N,S-C/In_2_O_3_ DHR possesses the strongest and broadest absorption in a visible-light region under the same conditions, and the optical absorption of other five samples follows the order of N,S-C/In_2_O_3_ NP > N-C/In_2_O_3_ NP > In_2_O_3_ DHR > In_2_O_3_ HD > commercial In_2_O_3_. This result indicates that N,S-codoped carbon layer and DHR structure play a synergistic role in enhancing the optical absorption ability of N,S-C/In_2_O_3_ DHR.

The separation and migration of electron-hole pairs is the second step of photocatalysis. In order to investigate this step, we measured the electrochemical impedance spectroscopy (EIS), photocurrent density, and linear sweep voltammetry (LSV) of N,S-C/In_2_O_3_ DHR, N,S-C/In_2_O_3_ NP, N-C/In_2_O_3_ NP, In_2_O_3_ DHR, In_2_O_3_ HD and commercial In_2_O_3_. The LSV curves (Fig. 4b) of these six samples indicate that the N,S-C/In_2_O_3_ DHR has the highest cathodic current density (CCD) for reducing H_2_O to H_2_. Moreover, the CCD of N,S-C/In_2_O_3_ NP is higher than that of N-C/In_2_O_3_ NP. We then compared the LSV curves of the three samples without the coated carbon layer (In_2_O_3_ DHR, In_2_O_3_ HD and commercial In_2_O_3_), and found that the In_2_O_3_ DHR showed higher CCD than In_2_O_3_ HD and commercial In_2_O_3_. Thus, it can be concluded that the N,S-codoped carbon layer and one-dimensional hollow rod structure are both beneficial to accelerate the charge transfer. As shown in Fig. 4c, the order of photocurrent density among these six samples is N,S-C/In_2_O_3_ DHR > N,S-C/In_2_O_3_ NP > N-C/In_2_O_3_ NP > In_2_O_3_ DHR > In_2_O_3_ HD > commercial In_2_O_3_, indicating that the N,S-C/In_2_O_3_ DHR also has the highest separation efficiency of photogenerated carriers. Their photoluminescence (PL) emission spectra (Supplementary Fig. 12) were recorded to prove the high-resolution rate of photogenerated carriers. The emission peak at 498 nm was assigned to the band-band PL phenomenon. The intensity of the peaks shows the order of N,S-C/In_2_O_3_ DHR < N,S-C/In_2_O_3_ NP < N-C/In_2_O_3_ NP < In_2_O_3_ DHR < In_2_O_3_ HD < commercial In_2_O_3_. The emission intensity of N,S-C/In_2_O_3_ DHR nearly vanish, meaning that the N,S-codoped carbon layer acts as the electron acceptor to effectively prevent the recombination of photogenerated carriers. This result is well consistent with the photocurrent density study. Figure 4d shows the electrochemical impedance spectra of these six samples. The N,S-C/In_2_O_3_ DHR presents the smallest semicircular curve in the Nyquist plot, indicating its lowest charge-transfer resistance, which is consistent with its more effective separation of photogenerated electrons and holes^47^. Therefore, N,S-codoped C layer evenly coated on In_2_O_3_ provides the electron storage and transfer channel to improve the separation efficiency of photogenerated carriers.

### Photocatalytic activity of the photocatalysts

**Fig. 5:**
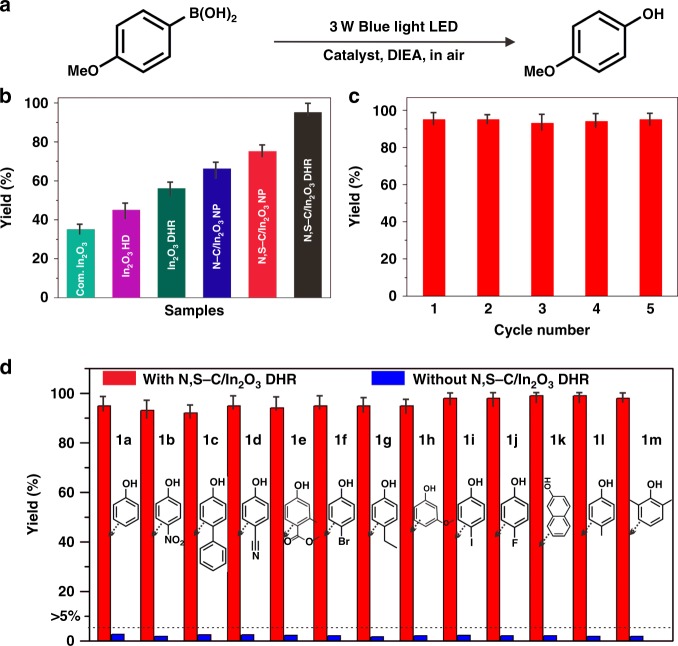
Photocatalytic oxidative hydroxylation. **a** Photocatalytic reaction for OHAA. **b** Reaction yields from OHAA using N,S-C/In_2_O_3_ DHR, N,S-C/In_2_O_3_ NP, N-C/In_2_O_3_ NP, In_2_O_3_ DHR, In_2_O_3_ HD, and commercial (com.) In_2_O_3_ as catalysts for 12 h. **c** Recycling tests for OHAA using N,S-C/In_2_O_3_ DHR as a catalyst under blue LED irradiation. **d** Reaction yields of various phenols obtained from oxidative hydroxylation of different arylboronic acids with and without N,S-C/In_2_O_3_ DHR as a photocatalyst. Source data are provided as a Source Data file

Photocatalytic activity of the as-prepared N,S-C/In_2_O_3_ DHR was evaluated by blue light LED induced oxidative hydroxylation of arylboronic acids (OHAA) to phenols. Phenols are very important intermediates for many polymers, natural products and pharmaceutical molecules^48–52^. The photocatalytic reactions were carried out using *N*,*N*-diisopropylethylamine (DIEA) as the sacrificing agent (Fig. 5a). The details for determining the optimized conditions for this photocatalytic reaction are shown in Supplementary Table 1. When compared with N,S-C/In_2_O_3_ NP, N-C/In_2_O_3_ NP, In_2_O_3_ DHR, In_2_O_3_ HD and commercial In_2_O_3_ (Fig. 5b and Supplementary Fig. 13), N,S-C/In_2_O_3_ DHR exhibits the highest catalytic activity with the production yield reaching about 99% after 12 h (determined by ^1^H NMR spectra, Supplementary Fig. 14). Compared to N-C/In_2_O_3_ HD (the In_2_O_3_ dodecahedron coated by N-doped C layer)^43^, the catalytic time of N,S-C/In_2_O_3_ DHR for achieving 99% production yield is reduced to half, indicating that the N,S-codoping plays an importantly positive role in improving the activity of carbon-coated photocatalysts. On the other hand, the photocatalyst is relatively less active under green and yellow light irradiation for 12 h, with the yields of 44 and 15%, respectively (Supplementary Fig. 15). The photocatalytic activity of other five samples follows the trend of N,S-C/In_2_O_3_ NP > N-C/In_2_O_3_ NP > In_2_O_3_ DHR > In_2_O_3_ HD > commercial In_2_O_3_, which is consistent with the analytic results shown in Fig. 4. Therefore, effective photocatalytic activity of N,S-C/In_2_O_3_ DHR should be assigned to the efficient separation of photogenerated carriers induced by uniform coating of the N,S-codoped C layer and enhanced optical absorption provided by the N,S-codoped C layer and DHR structure.

In order to access the recycling stability of N,S-C/In_2_O_3_ DHR, the same photocatalytic OHAA was carried out for five cycles with an interval of 12 h. The N,S-C/In_2_O_3_ DHR exhibits high recycling stability and stable photocatalytic efficiency (Fig. 5c). The shape and structure of the N,S-C/In_2_O_3_ DHR did not show obvious changes after photocatalytic cycle experiments (Supplementary Fig. 16). After keeping the solutions for 12 h, N,S-C/In_2_O_3_ DHR precipitated to the bottom of solution, while N,S-C/In_2_O_3_ NP still suspended in the solution (Supplementary Fig. 17). Therefore, the N,S-C/In_2_O_3_ DHR can be recovered from the solution through simple centrifugation. We also measured the dispersity of N,S-C/In_2_O_3_ DHR in the reaction solution. N,S-C/In_2_O_3_ DHR can be well dispersed in the solution under continuous stirring (Supplementary Fig. 18). The catalytic activity of the N,S-C/In_2_O_3_ DHR for OHAA with various substrates was also investigated under the same catalytic experimental conditions (Fig. 5d). The corresponding reaction yields of the products are about 90–95%, indicating that the efficient photocatalytic activity of N,S-C/In_2_O_3_ DHR is not specific to only one reaction, but highly suitable for a series of OHAA.

### Photocatalytic mechanism for OHAA

In order to understand the photocatalytic mechanism using N,S-C/In_2_O_3_ DHR as the photocatalyst, the energy band position of In_2_O_3_ was first calculated. As show in Supplementary Fig. 19, the absorption edge presents at 443 nm in visible-light region. The bandgap of the In_2_O_3_ DHR was estimated to 2.8 eV, matching well with the reported value^53^. Thus, the In_2_O_3_ DHR works well under blue LED light irradiation (*E* = 2.76 ± 4 eV)^54^. The VB potential of In_2_O_3_ can be calculated by Mulliken electronegativity theory:

1 $$E_{\mathrm{VB}} = X-E^e + 0.5E_{\mathrm{g}}$$

where *E*_VB_ is corresponding VB potential, *X* is the absolute electro-negativity of corresponding semiconductor, *E*^*e*^ is the energy of free electrons in the hydrogen scale (about 4.5 eV), and *E*_g_ is the bandgap energy of corresponding semiconductor. The *X* value for In_2_O_3_ is 5.19 eV, and thus the *E*_VB_ value of In_2_O_3_ is calculated to be 2.09 eV. The *E*_VB_ value was also confirmed by the VB XPS (Supplementary Fig. 20). The CB potential (*E*_CB_) can be obtained by:

2 $$E_{\mathrm{CB}} = E_{\mathrm{VB}}-E_{\mathrm{g}}$$

In order to reflect the effect of pH, the following equation was used to calculate the *E*_VB_ and *E*_CB_ potentials of the photocatalyst:

3 $$E = E^0-0.05915 \times {\mathrm{pH}}$$

Thus, the *E*_VB_ and *E*_CB_ values of In_2_O_3_ at pH = 7 were estimated to be 1.68 and −1.12 V vs normal hydrogen electrode (NHE), respectively.

**Fig. 6:**
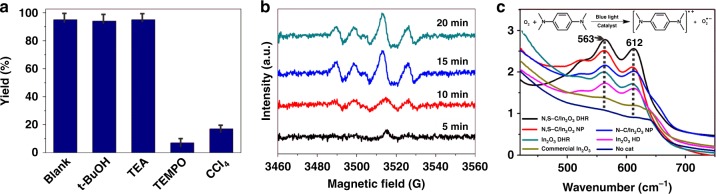
Photocatalytic mechanism studies. **a** Control experiments for OHAA over N,S-C/In_2_O_3_ DHR under blue LED irradiation in the presence of different scavengers: no scavenger (blank), t-BuOH, TEA, TEMPO, and CCl_4_. **b** EPR spectra of a reaction solution in the present of DMPO under blue LED irradiation for different times. **c** UV–vis absorption spectra for cationic radicals of TMPD treated by N,S-C/In_2_O_3_ DHR, N,S-C/In_2_O_3_ NP, N-C/In_2_O_3_ NP, In_2_O_3_ DHR, In_2_O_3_ HD, and commercial In_2_O_3_ under blue LED light irradiation. Source data are provided as a Source Data file

A series of comparative experiments were performed by adding various radical scavengers into the reaction system to investigate the major reactive species in the photocatalytic OHAA. Tertiary butanol (t-BuOH) was adopted to extinguish hydroxyl radical (∙OH), triethylamine (TEA) was introduced to quench photogenerated hole (h^+^), (2,2,6,6-tetramethylpiperidin-1-yl)oxidanyl (TEMPO) was employed to scavenge superoxide anion radical (∙O_2_^−^), and CCl_4_ was used for trapping photogenerated electron (e^−^)^55–57^. As illustrated in Fig. 6a, before and after adding TEA and t-BuOH, the yield of corresponding phenol remains almost unchanged (Supplementary Figures 21–23). When TEMPO is added to quench ∙O_2_^−^, the yield of phenol is significantly suppressed (Supplementary Figure 24). Noticeable inhibition can be also observed when CCl_4_ is added to trap e^−^ (Supplementary Figure 25). These results reveal that the e^-^ and ∙O_2_^−^ play the key roles in OHAA using N,S-C/In_2_O_3_ DHR as the photocatalyst. Electron paramagnetic resonance (EPR) was used to prove the existence of ∙O_2_^−^, which was captured by adding 5,5-dimethyl-1-pyrroline-N-oxide (DMPO). Obvious characteristic signals of ∙O_2_^− ^can be detected when DMPO is used as the radical scavenger, and the intensity of the signals gradually increases upon the illumination time (Fig. 6b). The results indicate that ∙O_2_^− ^is the primary active intermediate species in this photocatalytic process.

The superoxide anion radical ∙O_2_^−^ can only be produced by reacting dissolved O_2_ with photogenerated electrons under the CB potential of O_2_/∙O_2_^−^ (−0.33 V vs NHE). In this system, the CB potential (−1.12 V vs NHE) of In_2_O_3_ is high enough to form ∙O_2_^−^ based on the thermodynamic viewpoint. The generation of ∙O_2_^−^ in the N,S-C/In_2_O_3_ DHR system was further verified by using N,N,N′,N′-tetramethyl-phenylenediamine (TMPD), because the photoactive systems can regulate the electrons transfer from TMPD molecule to O_2_, resulting in the production of ∙O_2_^−^ and blue-colored product with obvious absorption peaks at 612 nm and 563 nm^58^. All of the six samples (N,S-C/In_2_O_3_ DHR, N,S-C/In_2_O_3_ NP, N-C/In_2_O_3_ NP, In_2_O_3_ DHR, In_2_O_3_ HD and commercial In_2_O_3_) produce characteristic absorption peaks after reacting with TMPD (Fig. 6c), indicating that In_2_O_3_ has the ability to generate ∙O_2_^−^. The characteristic absorption peak intensity of these six samples follows the order of N,S-C/In_2_O_3_ DHR > N,S-C/In_2_O_3_ NP > N-C/In_2_O_3_ NP > In_2_O_3_ DHR > In_2_O_3_ HD > commercial In_2_O_3_, which is consistent with the trend of the separation and migration ability of photogenerated carriers. The high-resolution O 1s spectra of these six In_2_O_3_ samples were measured to investigate the surface structures of photocatalysts (Supplementary Fig. 26 and Supplementary Table 2). Relative proportion of the oxygen vacancy (O_V_) component in these samples is in the order of N,S-C/In_2_O_3_ DHR > N,S-C/In_2_O_3_ NP > N-C/In_2_O_3_ NP > In_2_O_3_ DHR > In_2_O_3_ HD > commercial In_2_O_3_, indicating that the O_V_ provides the site for activating O_2_ to ∙O_2_^−^. In the N,S-C/In_2_O_3_ DHR system, ∙O_2_^−^ is the main reactive species for photocatalytic OHAA, and e^−^ is responsible for reducing the oxygen molecule to produce ∙O_2_^−^.

**Fig. 7:**
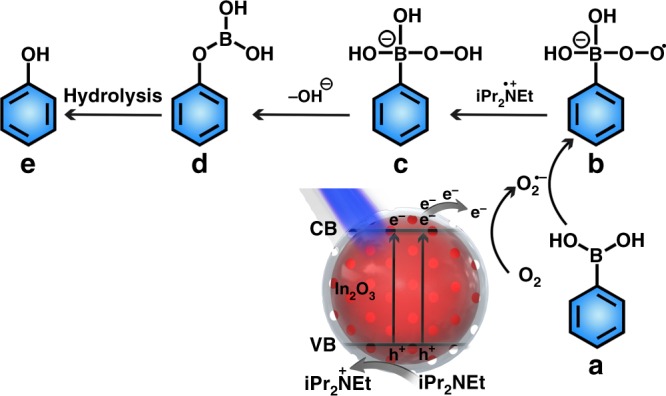
Schematic illustration exhibiting the proposed reaction mechanism (a–e) for OHAA using N,S-C/In_2_O_3_ DHR as the photocatalyst

Based on above studies, a possible photocatalytic mechanism of OHAA over N,S-C/In_2_O_3_ DHR was proposed (Fig. 7). Firstly, the photogenerated carriers are excited in N,S-C/In_2_O_3_ DHR under blue LED irradiation. Then, the photogenerated electrons are transferred to the N,S-codoped carbon layer, and the photogenerated holes still stay on the In_2_O_3_ nanoparticles. Secondly, the adsorbed O_2_ molecule is reduced by separated electrons, resulting in the formations of ∙O_2_^−^. The DIEA molecule (iPr_2_Net) can capture the separated holes to form iPr_2_N^+^Et. Thirdly, the obtained ∙O_2_^−^ would react with arylboronic acid molecule (a) to generate intermediate (b). The intermediate (b) then abstracts a hydrogen atom from iPr_2_N^+^Et to form intermediate (c), which is further rearranged with the loss of a –OH^−^ ion to form (d). The intermediate (d) is subsequently hydrolyzed to afford the final phenol product (e).

**Fig. 8:**
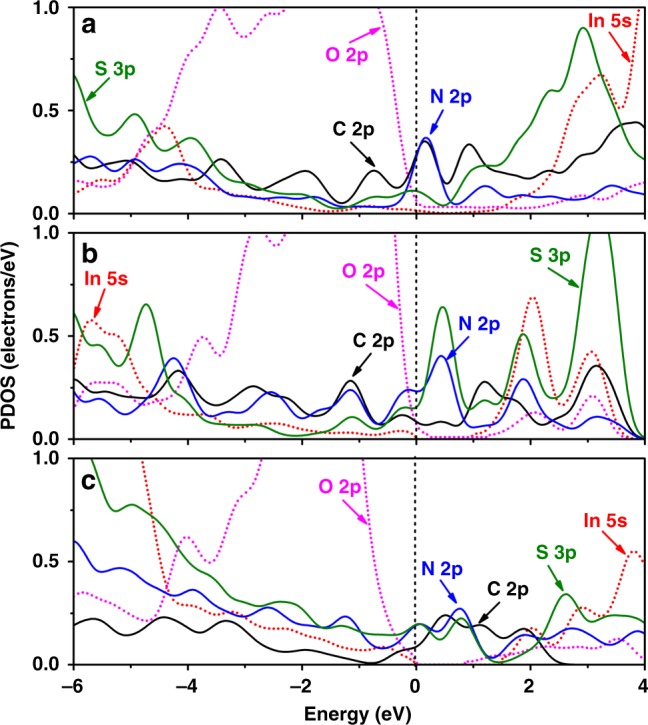
Density functional theory calculations. PDOS for **a** In_2_O_3_ (100) face/N,S-codoped graphite (001) face, **b** In_2_O_3_ (110) face/N,S-codoped graphite (001) face, and **c** In_2_O_3_ (111) face/N, S-codoped graphite (001) face

Microcosmic mechanism of photogenerated carrier separation and transfer on N,S-codoped In_2_O_3_ was further investigated by calculating the projected density of states (PDOS) from In_2_O_3_ (001) face/N,S-codoped graphite (001) face, In_2_O_3_ (110) face/N,S-codoped graphite (001) face, and In_2_O_3_ (111) face/N,S-codoped graphite (001) face, respectively. As shown in Fig. 8, the hybrid states of C 2p orbital, S 3p orbital, and N 2p orbital cross over the bandgap of In_2_O_3_ in the three In_2_O_3_/N,S-codoped graphite interfaces, meaning that the electrons from O 2p state of In_2_O_3_ would be transferred to these hybrid states of graphite. Moreover, the hybridization of N 2p, S 3p, and C 2p increases the strength of interstitial states to further improve the separation and transfer rate of photogenerated carriers. Thus, electrons are mainly excited from O 2p state of In_2_O_3_ to hybrid states of graphite (N 2p, S 3p, and C 2p orbitals) through the interface, leaving the photogenerated holes on O 2p state of In_2_O_3_.

## Discussion

In conclusion, the double-shelled hollow rods (N,S-C/In_2_O_3_ DHR) assembled by N,S-codoped C coated In_2_O_3_ ultrafine nanoparticles have been synthesized by thermolysis of In-based frameworks. The obtained N,S-C/In_2_O_3_ DHR possesses effective catalytic activity for photocatalytic OHAA with high stability and easy recovery at the same time. The excellent performance of N,S-C/In_2_O_3_ DHR in the photocatalysis is due to the following four structural characteristics. Firstly, the hollow rods not only provide a local microenvironment for OHAA, but also enhance the light absorption by increasing the optical reflection in the hollow interior to generate more electrons and holes. Secondly, the double shells of N,S-C/In_2_O_3_ DHR have lots of reactive sites, which improve the photocatalytic activity. Thirdly, the mesoporous structure of the shells improves the accessibility of N,S-C/In_2_O_3_ DHR, which is beneficial for the diffusion of reactants and products. Fourthly, the regular morphology and large size (6–7 µm in length) of N,S-C/In_2_O_3_ DHR significantly improve the recyclability and reusability. The photocatalytic mechanism of OHAA over N,S-C/In_2_O_3_ DHR has been proposed, confirming that ∙O_2_^−^ is the primary reactive species in this catalytic reaction. In addition, the microcosmic separation and transfer pathways via the interface of In_2_O_3_/N,S-codoped graphite have been clarified by PDOS theoretical calculations, i.e., photogenerated electrons are mainly excited from O 2p state of In_2_O_3_ and transferred to hybrid states of the graphite consisting of S 3p, N 2p, and C 2p orbitals, leaving photogenerated holes on O 2p state of In_2_O_3_. Thus, this work presents useful information on the rational exploration of In_2_O_3_-based hybrid materials for achieving highly efficient photocatalysts.

## Methods

### Synthesis of N,S-codoped MIL-68-In

Indium(III) nitrate hydrate (0.21 mmol, 0.08 g), 1,4-benzenedicarboxylic acid (0.18 mmol, 0.03 g) and BIT (0.13 mmol, 0.02 g) were separately added into N,N-dimethylformamide (10 mL). After ultrasonication for 10 min, the above solution was directly transferred to a Teflon-lined steel autoclave (50 mL) and put in oven at 100 °C under static conditions for 4 h. When the reaction completed, the sample was obtained by centrifugation, washed with ethanol for several times, and finally dried at 60 °C for 24 h.

### Synthesis of N,S-C/In_2_O_3_ DHR

N,S-C/In_2_O_3_ DHR was obtained via the pyrolysis of the N,S-codoped MIL-68-In precursor in a vacuum atmosphere at 550 °C with the heating rate of 5 °C min^−1^ for 45 min.

### Synthesis of In_2_O_3_ DHR

In_2_O_3_ DHR was synthesized via the pyrolysis of the obtained N,S-C/In_2_O_3_ DHR in O_2_ at 550 °C with the heating rate of 5 °C min^−1^ for 45 min.

### Synthesis of N,S-C/In_2_O_3_ NP

Indium(III) nitrate hydrate (0.21 mmol, 0.08 g), benzimidazole (0.25 mmol, 0.03 g), 1,4-benzenedicarboxylic acid (0.18 mmol, 0.03 g) and BIT (0.13 mmol, 0.02 g) were dissolved in methanol (10 mL). After ultrasonication for about 10 min, the solution was directly poured into a Teflon-lined steel autoclave (50 mL), and put in an oven at 100 °C under static conditions for 240 min. When the reaction finished, the product was obtained through the centrifugation, which was washed several times with ethanol and finally dried at 60 °C for 24 h. N,S-C/In_2_O_3_ NP was obtained by the pyrolysis of the product in a vacuum environment at 450 °C with the heating rate of 2 °C min^−1^ for 1 h.

### Synthesis of N-C/In_2_O_3_ NP

Indium(III) nitrate hydrate (0.21 mmol, 0.08 g), benzimidazole (0.254 mmol, 0.03 g) and 1,4-benzenedicarboxylic acid (0.18 mmol, 0.03 g) were separately added into methanol (10 mL). After ultrasonication for about 10 min, the solution was directly poured into a Teflon-lined steel autoclave (50 mL), and put in the oven at 100 °C under static conditions for 240 min. When the reaction finished, the product was obtained by centrifugation, washed with ethanol for several times, and finally dried at 60 °C for 24 h. N-C/In_2_O_3_ NP was obtained by the pyrolysis of the product in a vacuum environment at 450 °C with the temperature increasing speed of 2 °C min^−1^ for 1 h.

### Synthesis of In_2_O_3_ HD

Indium(III) nitrate hydrate (0.040 mmol, 0.015 g), benzimidazole (0.846 mmol, 0.1 g) and 4,5-imidazoledicarboxylic acid (0.135 mmol, 0.021 g) were separately dissolved in N,N-dimethylformamide (6 mL). After ultrasonication for about 10 min, the solution was directly transferred to a round bottom flask (50 mL) heated at 100 °C for 4 h. When the reaction finished, the sample was obtained by centrifugation, washed with ethanol for several times, and then dried at 60 °C for 24 h. The pyrolysis was performed on the sample in an argon environment at 500 °C with the heating rate about 2 °C min^−1^ for 1 h to afford product A. In_2_O_3_ HD was obtained via the pyrolysis of the product A in an oxygen environment at 500 °C with a heating rate about 2 °C min^−1^ for 6 h.

### Characterizations

The compositions and phases of the obtained samples were characterized by powder XRD on the PANalytical X’Pert diffractometer with a CuKα radiation. SEM (SU8100) and HRTEM (FEI Tecnai-F20) were used to characterize the shape and crystal structure. The surface compositions of samples were characterized by PHI QUANTUM2000 photoelectron spectrometer (XPS). N_2_ adsorption/desorption isotherms were used to characterize the surface areas of samples based on the BET method (Micrometrics ASAP 2020 system). Bruker ESP-300E spectrometer was used to measure EPR spectra at 9.8 GHz with X-band and 100 Hz field modulation. Dynamic light scattering (DLS) experiments were conducted on a Nano-Zetasizer (Nano-ZS) from Malvern Instruments (Malvern, UK). The experimental process was as follows: samples (0.1 g) and ethanol (10 mL) were added into a glass bottle (15 mL) and dispersed uniformly by ultrasonication for 10 min. Solutions were placed at different times under agitation and non-agitation, respectively. Then, the upper liquids were taken for DLS tests.

### Photoelectrochemical measurements

Photoelectrochemical experiments were measured in the three electrode quartz cell. The Pt plate was selected as the counter electrode, the reference electrode was Hg/HgCl_2_ electrode, and the corresponding working electrode was obtained on the fluorine doped tin oxide (FTO) glass. In order to obtain a slurry, the obtained product (10 mg) was ultrasonicated in ethanol (0.2 mL). The slurry was then spread onto the FTO glass with the other side protected by using Scotch tape. After drying in air, the Scotch tape was unstuck. In order to improve the adhesion, the obtained working electrode with the area about 2.5 cm^2^ was dried at 300 °C for 180 min. The CHI-760E workstation was used to measure the EIS. In the three-electrode cell, the 0.025 M KH_2_PO_4_ and 0.025 M Na_2_HPO_4_ standard buffer solution (25 °C, pH = 6.864) were selected as the electrolytes without adding any additive, and the measurements were performed on an open circuit potential condition. A 300 W Xe arc lamp system was used as the visible-light irradiation source. The LSV technique was employed to obtain cathodic polarization curves.

### Photocatalytic reactions

The photocatalyst (5 mg), DIEA (0.3 mmol), and phenylboronic acid (0.1 mmol, 1 equivalent) were separately dissolved into ethanol (2 mL). The stirring solution was irradiated under the blue LED (3 W, 450 nm) at room temperature in air. The yield of the product was characterized by ^1^H NMR spectra. To perform the recycling experiments, the photocatalyst was recovered by centrifugation and washed with dichloromethane for several times. The recycled photocatalyst was then dried in vacuum at about 60 °C for 24 h.

### Density functional calculations

Plane-wave pseudopotential method was used to calculate the density functional, which was implemented in the Cambridge Sequential Total Energy Package (CASTEP) code^59^. The exchange-correlation effects and electron-ion interactions were described by the local density approximation^60^ and ultrasoft pseudopotential^61^, respectively. A Morkhost-Pack mesh^62^ of *Γ* and 2 × 2 × 1 point were used to calculated geometry optimization and electronic properties, respectively. The self-consistent convergence accuracy, the convergence criterion for the force between atoms and the maximum displacement were set at 2 × 10^−6^ eV/atom, 5.5 × 10^−2^ eV/Å, and 2 × 10^−3^ Å, respectively.

### Reporting summary

Further information on research design is available in the Nature Research Reporting Summary linked to this article.

## Supplementary information

Supplementary Information

Peer Review File

Reporting Summary

Source Data

## Data Availability

The authors declare that the main data supporting the findings of this study are available within the paper and its Supplementary Information. The source data underlying Figs. 2, 3, 4, 5b–d and 6a–c and Supplementary Figs. 1a,i,q, 2a,b, 4, 5, 7–16, and 19–25 are provided as a Source Data file.
