# Supplementary material for: Double-shelled hollow rods assembled from nitrogen/sulfur-codoped carbon coated indium oxide nanoparticles as excellent photocatalysts
Source: Nat Commun. 2019 May 22;10:2270. doi: 10.1038/s41467-019-10302-0 (PMC6531469; doi:10.1038/s41467-019-10302-0)
Supplement: Supplementary file 1 — Supplementary Information [file 41467_2019_10302_MOESM1_ESM.pdf]

Supplementary Information

Double-shelled hollow rods assembled from nitrogen/sulfur-codoped carbon coated indium oxide nanoparticles as excellent photocatalysts

Sun *et al.*

Supplementary Figures

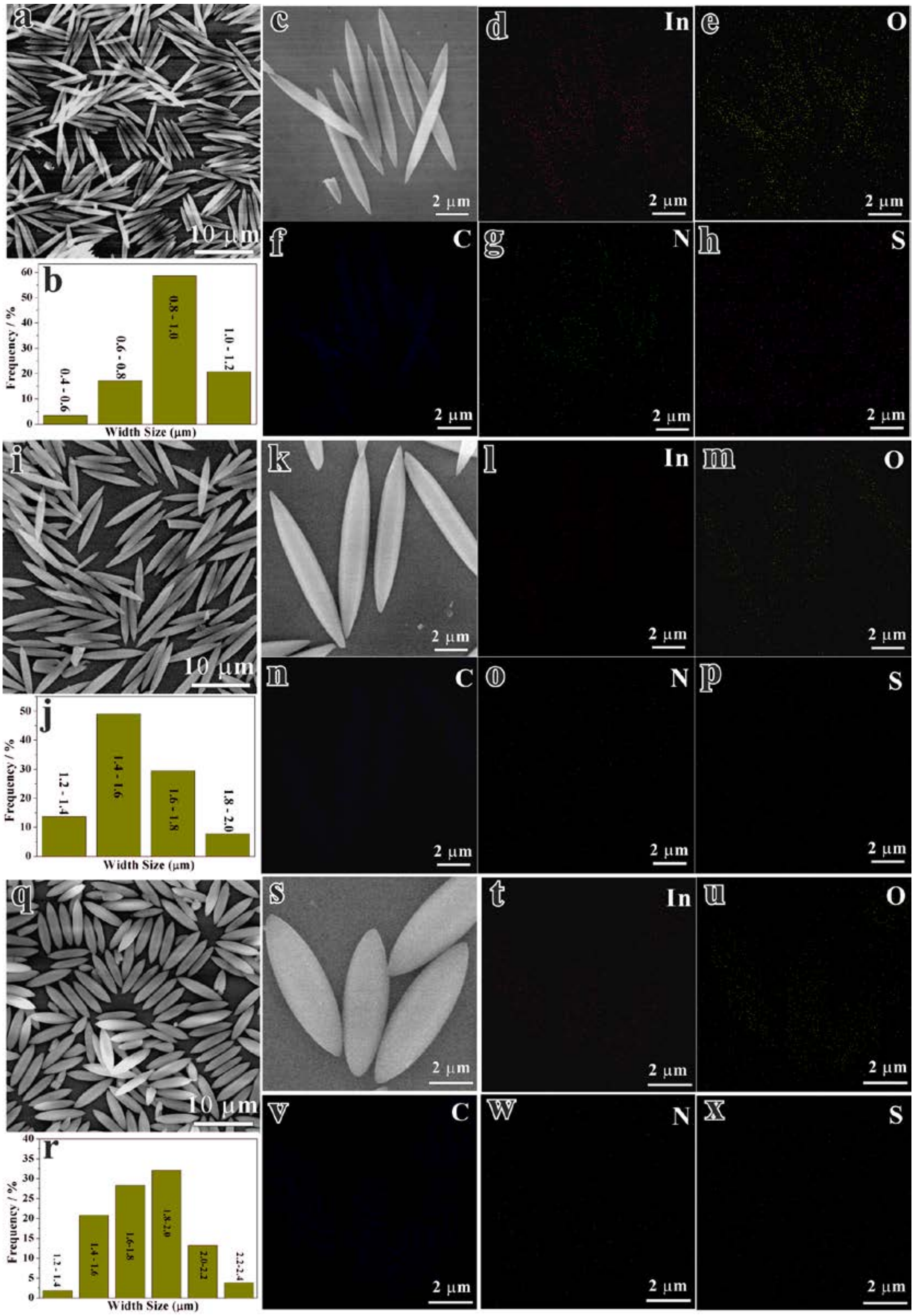

Supplementary Figure 1. Preparation of N,S-codoped MIL-68-In rods. N,S-codoped MIL-68-In rods with different widths prepared by using different amounts of BIT: (a-h) using 0.13 mmol (thin rods), (i-p) using 0.25 mmol (medium rods), and (q-x) using 0.36 mmol (thick rods). Source data are provided as a Source Data file.

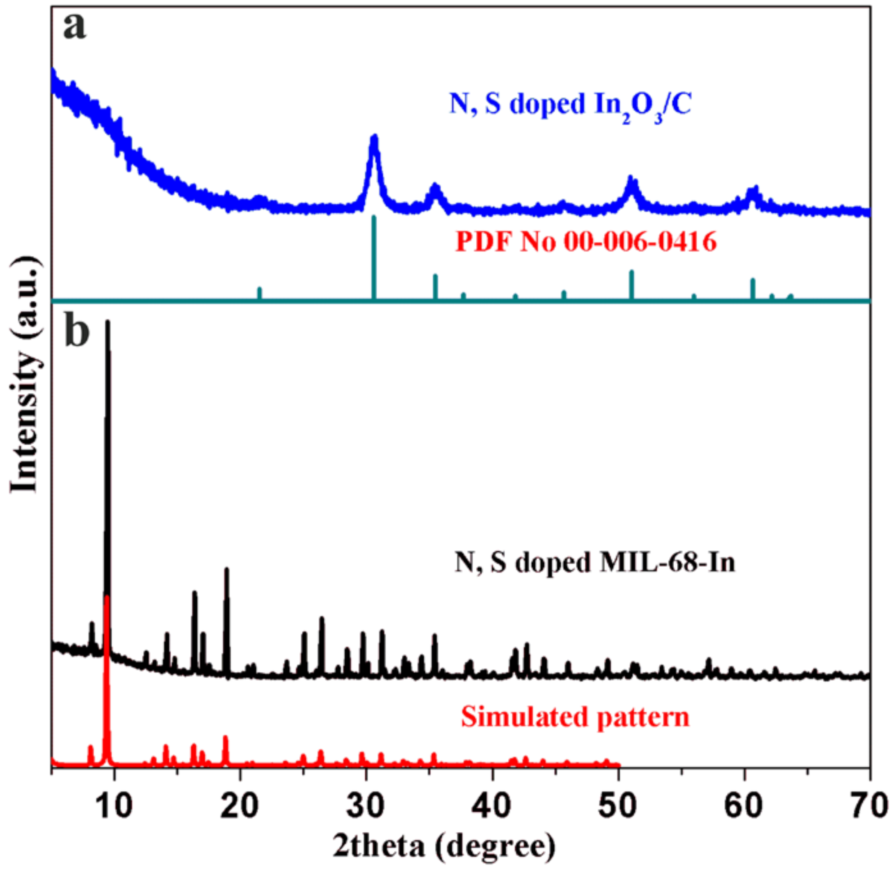

Supplementary Figure 2. Powder XRD patterns. (a) N,S-codoped MIL-68-In and (b) annealing product. Source data are provided as a Source Data file.

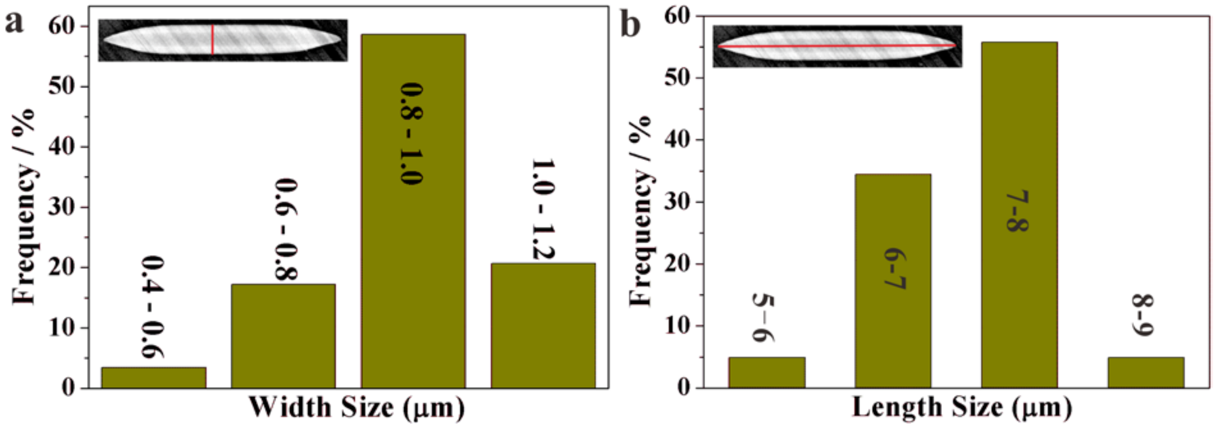

Supplementary Figure 3. Size distributions. (a,b) Sizes of N,S-codoped MIL-68-In.

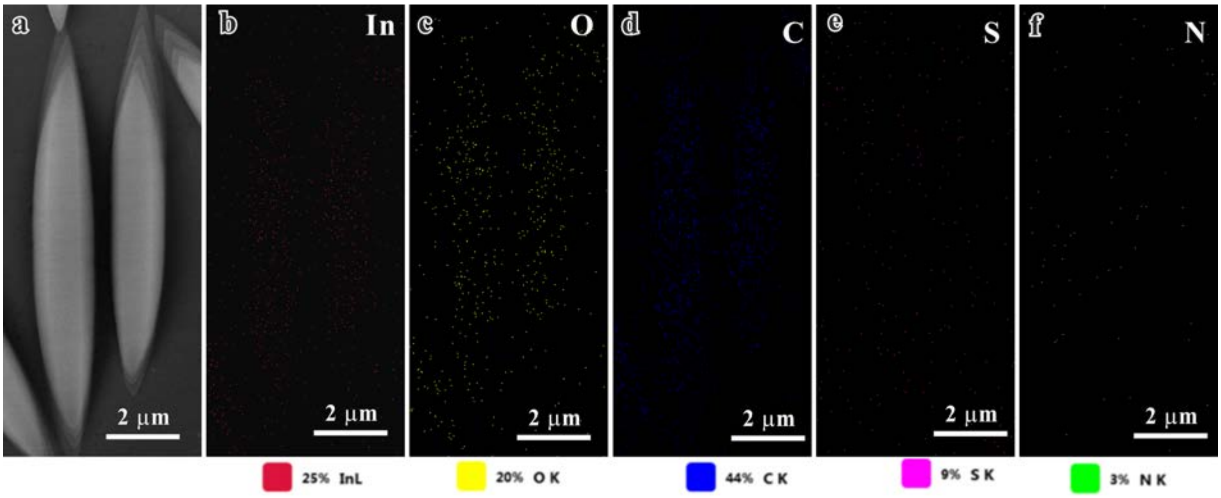

Supplementary Figure 4. Elemental mapping. (a-f) Corresponding elemental mapping of N,S-codoped MIL-68-In. Source data are provided as a Source Data file.

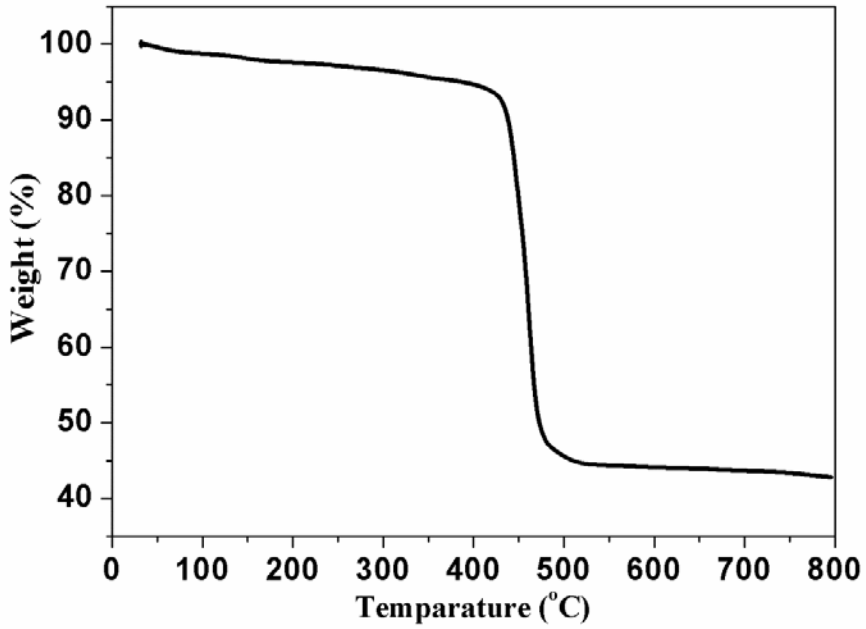

Supplementary Figure 5. TGA curve of the as-obtained N,S-codoped MIL-68-In. Source data are provided as a Source Data file.

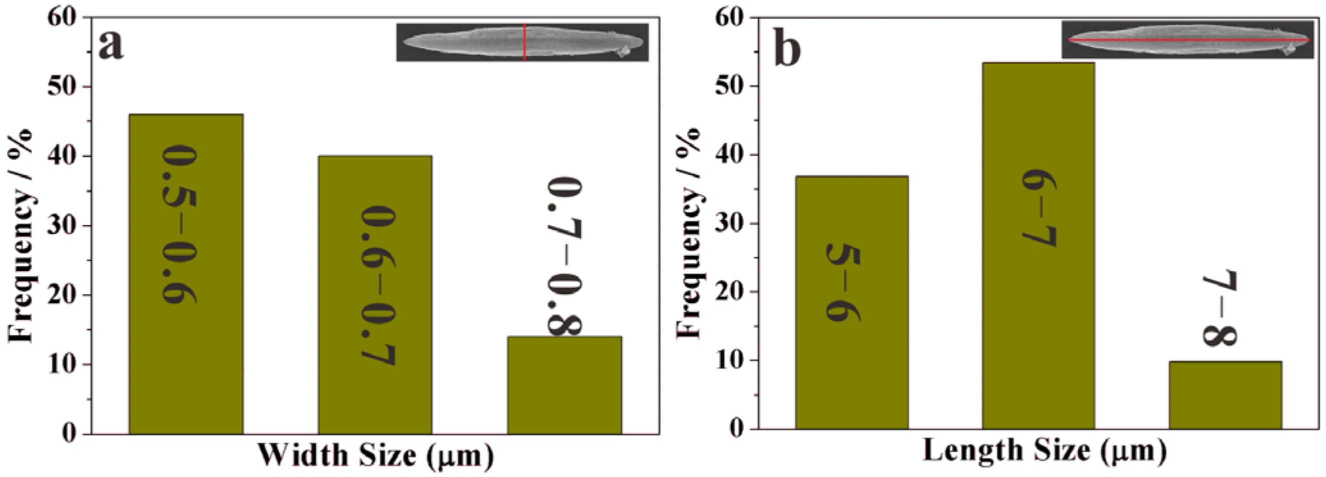

Supplementary Figure 6. Size distributions. (a,b) Sizes of N,S-C/In_2O_3 DHR.

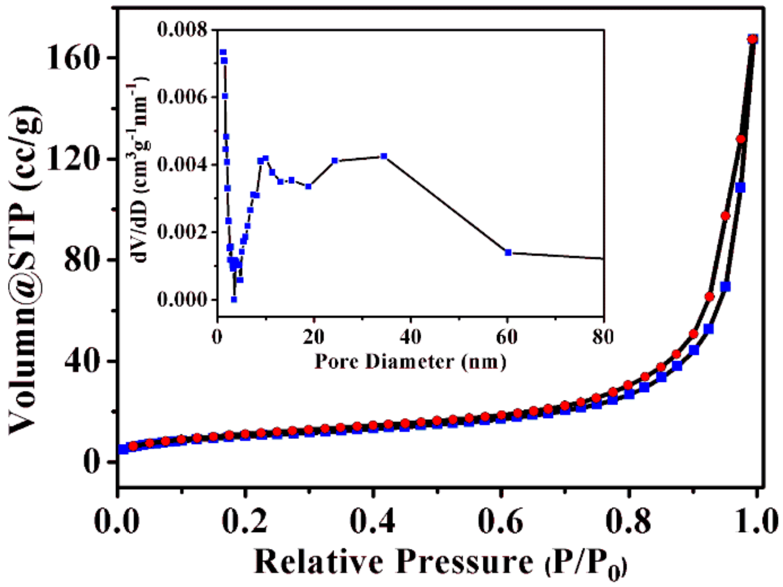

Supplementary Figure 7. N_2 adsorption/desorption isotherm and corresponding pore size distribution (inset) of N,S-C/In_2O_3 DHR. Source data are provided as a Source Data file.

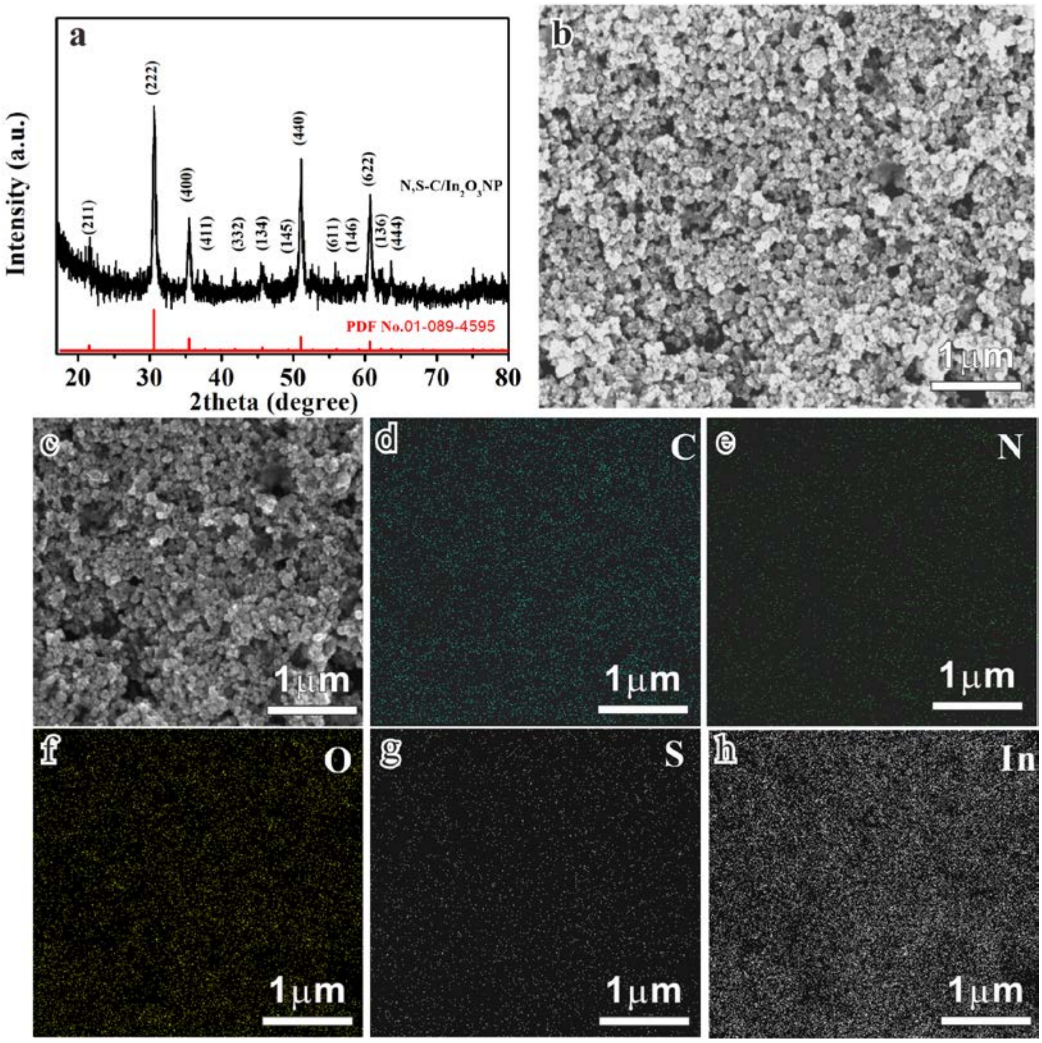

Supplementary Figure 8. Characterization of N,S-C/In_2O_3 NP. (a) Powder XRD pattern of N,S-C/In_2O_3 NP, (b) SEM image of N,S-C/In_2O_3 NP, and (c-h) corresponding elemental mapping. Source data are provided as a Source Data file.

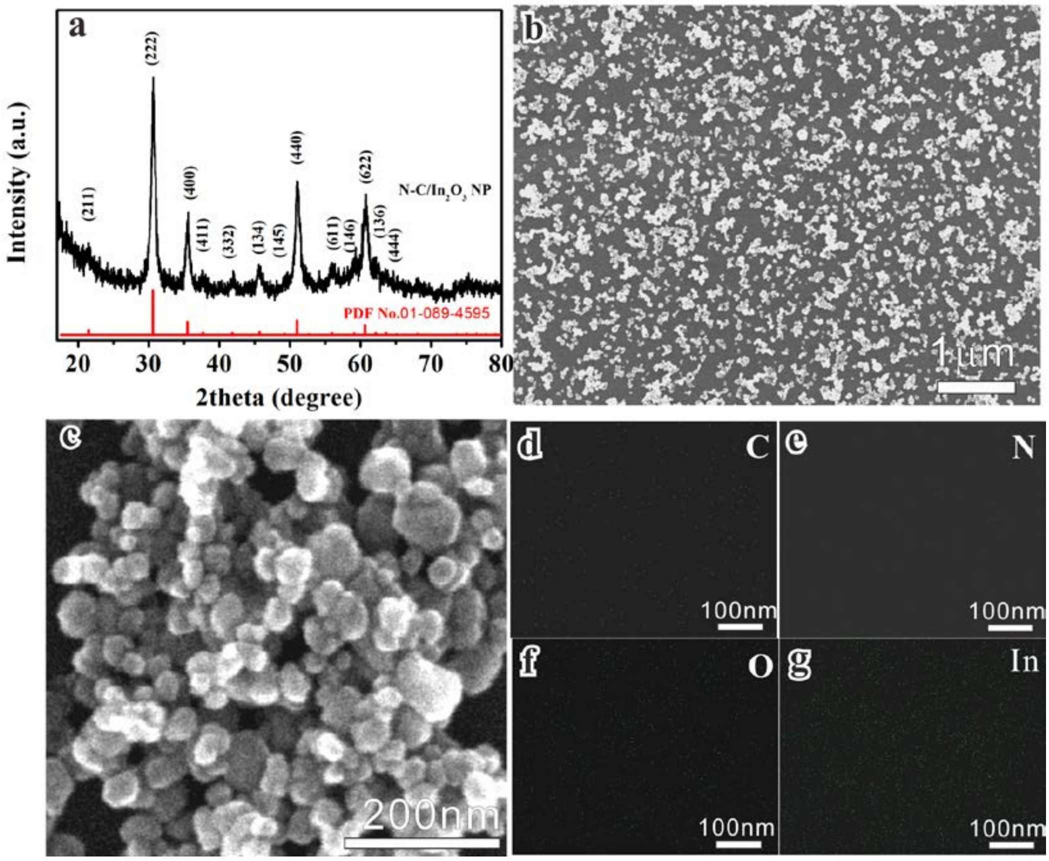

Supplementary Figure 9. Characterization of N-C/In_2O_3 NP. (a) Powder XRD pattern of N-C/In_2O_3 NP, (b) SEM image of N-C/In_2O_3 NP, and (c-g) corresponding elemental mapping. Source data are provided as a Source Data file.

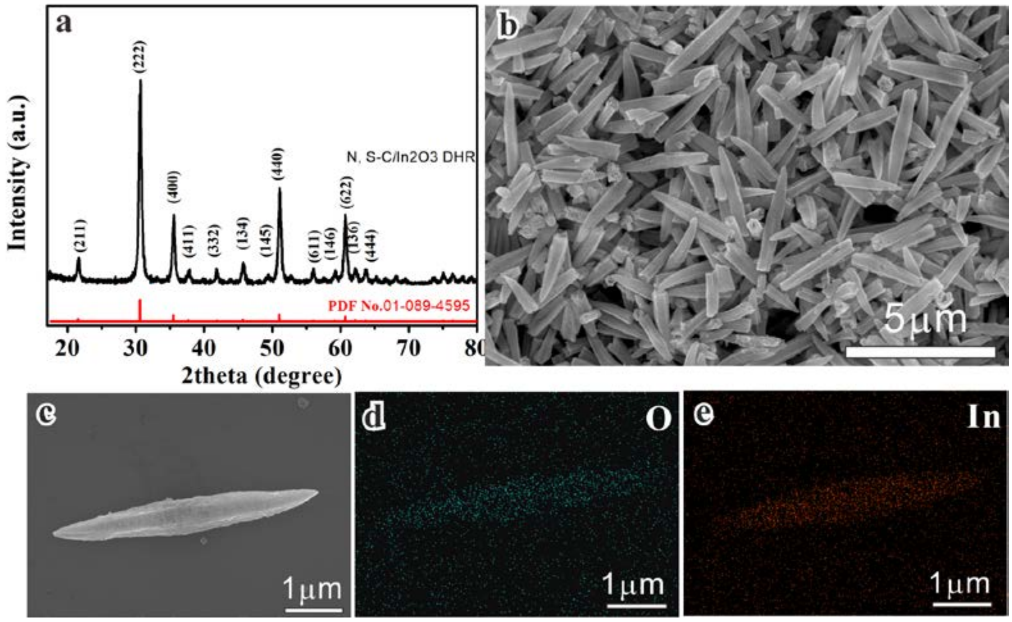

Supplementary Figure 10. Characterization of In_2O_3 DHR. (a) Powder XRD pattern of In_2O_3 DHR, (b) SEM image of In_2O_3 DHR, and (c-e) corresponding elemental mapping. Source data are provided as a Source Data file.

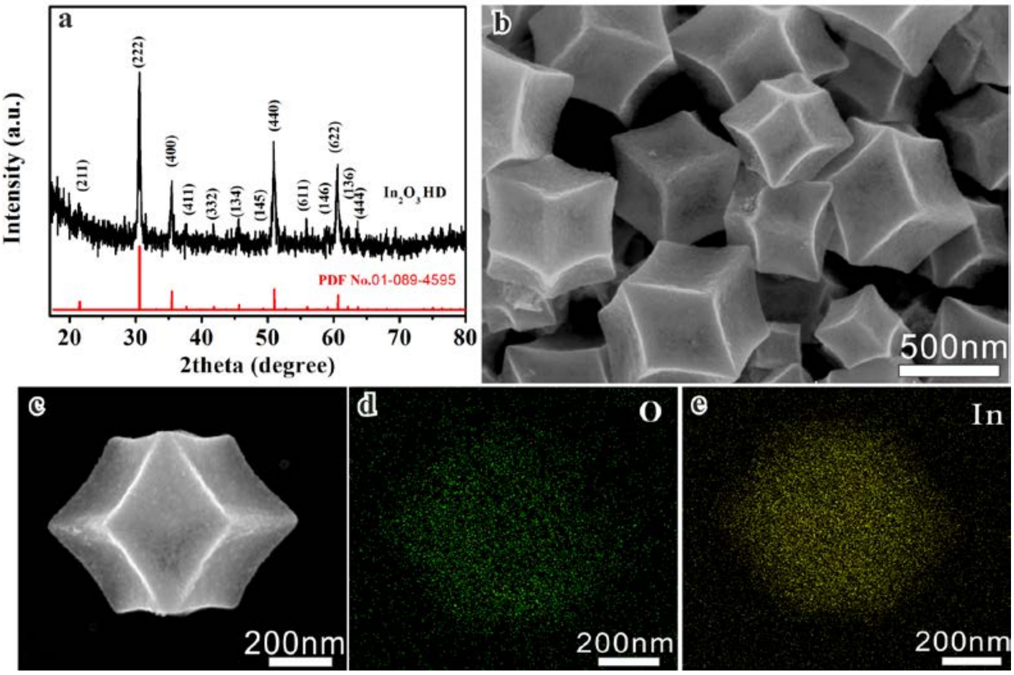

Supplementary Figure 11. Characterization of In_2O_3 HD. (a) Powder XRD pattern of In_2O_3 HD, (b) SEM image of In_2O_3 HD, and (c-e) corresponding elemental mapping. Source data are provided as a Source Data file.

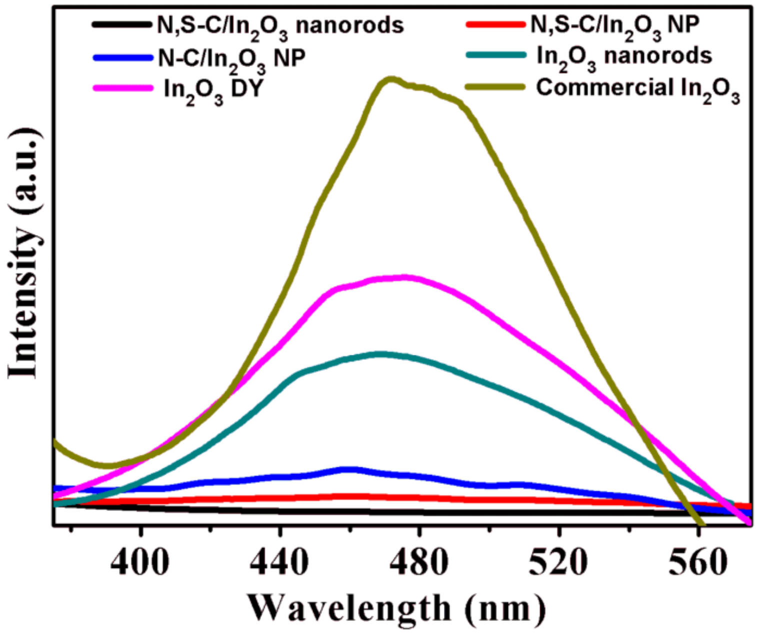

Supplementary Figure 12. Photoluminescence spectra of N,S-C/In_2O_3 DHR, N,S-C/In_2O_3 NP, N-C/In_2O_3 NP, In_2O_3 DHR, In_2O_3 HD, and commercial In_2O_3. Source data are provided as a Source Data file.

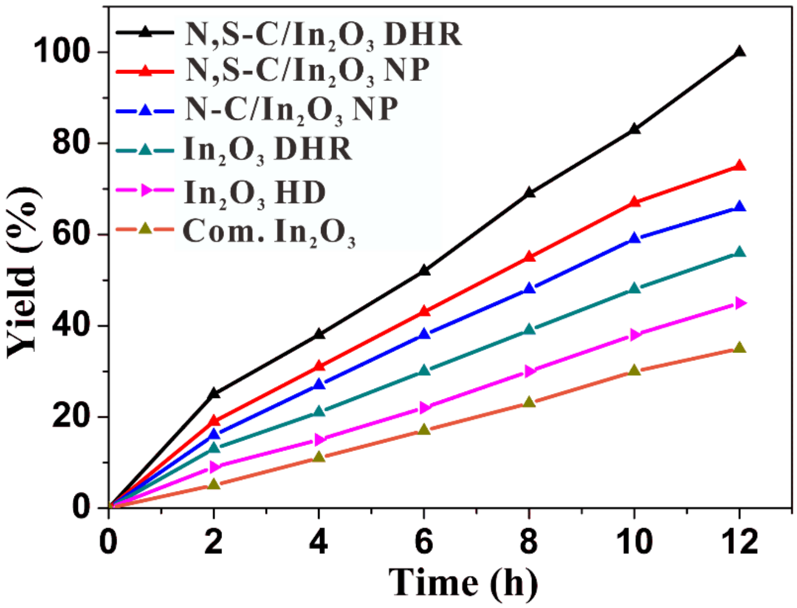

Supplementary Figure 13. Time courses of oxidative hydroxylation of phenylboronic acid using N,S-C/In_2O_3 DHR, N,S-C/In_2O_3 NP, N-C/In_2O_3 NP, In_2O_3 DHR, In_2O_3 HD and commercial In_2O_3 as the catalyst, respectively. Source data are provided as a Source Data file.

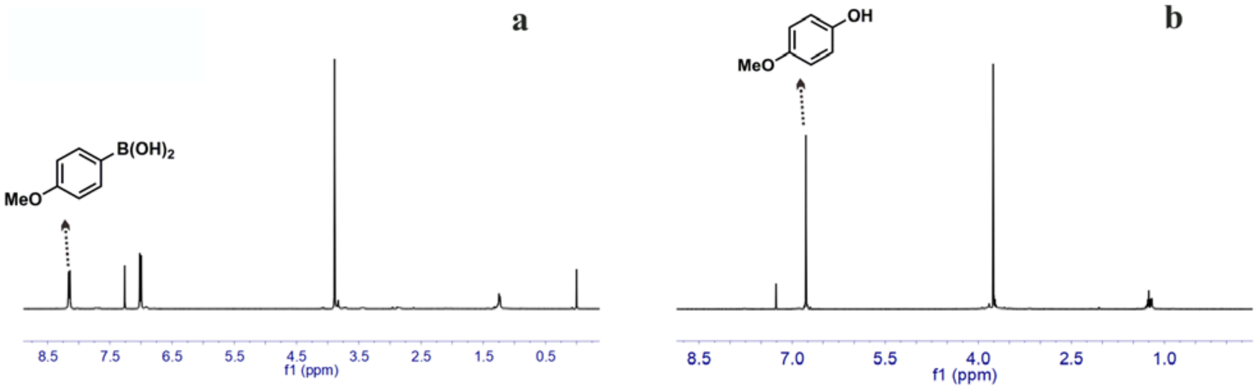

Supplementary Figure 14. ^{1}H NMR spectra. (a) ^{1}H NMR spectrum of the reactant, and (b) ^{1}H NMR spectrum of the product. Source data are provided as a Source Data file.

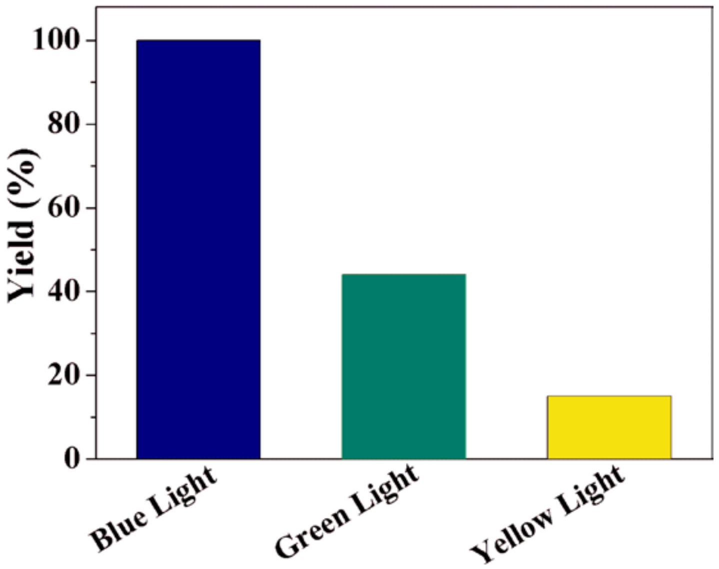

Supplementary Figure 15. Yields from oxidative hydroxylation of phenylboronic acid using N,S-C/In_2O_3 DHR under different LED light irradiation. Source data are provided as a Source Data file.

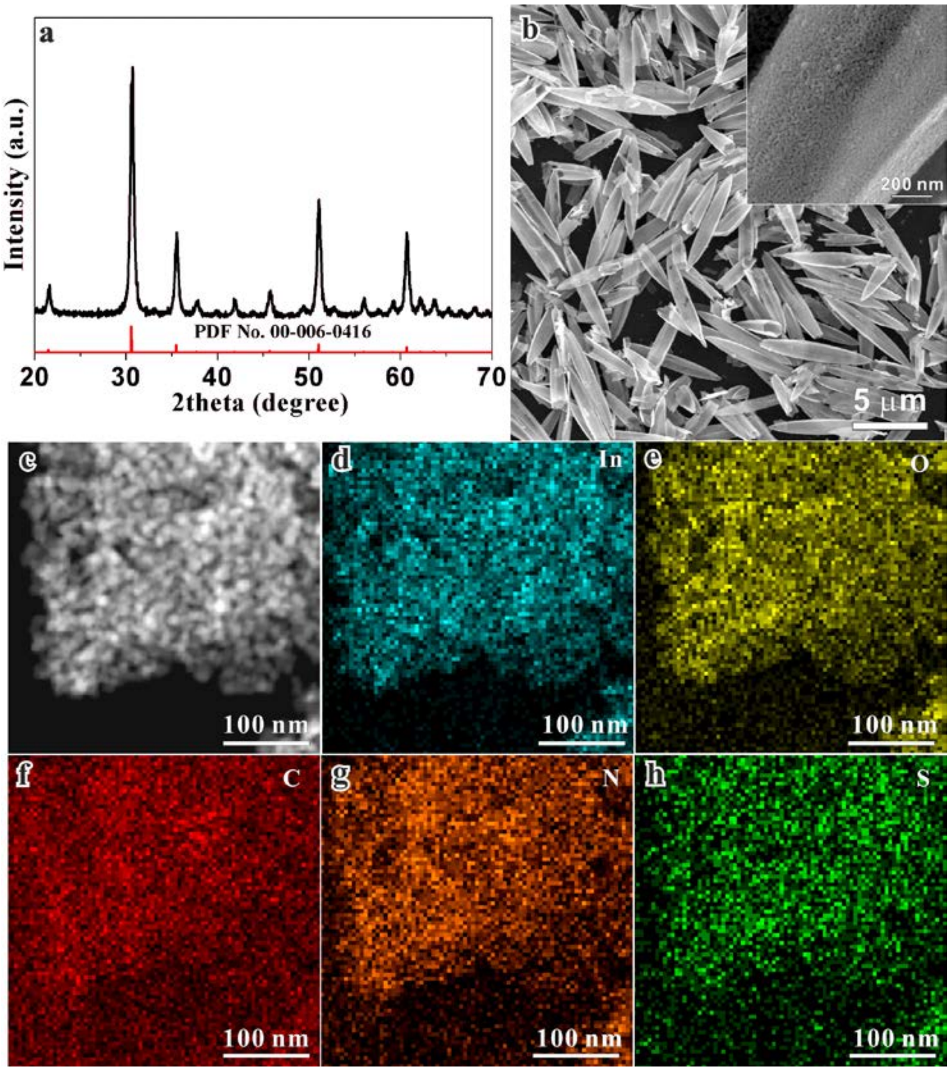

Supplementary Figure 16. Characterization of N,S-C/In_2O_3 DHR after five-cycle catalytic reaction. (a) Powder XRD pattern of N,S-C/In_2O_3 DHR after five-cycle catalytic reaction, (b) SEM image of N,S-C/In_2O_3 DHR after five-cycle catalytic reaction, and (c-h) STEM image and EDX elemental mapping of In, O, C, N, and S after five-cycle catalytic reaction. Source data are provided as a Source Data file.

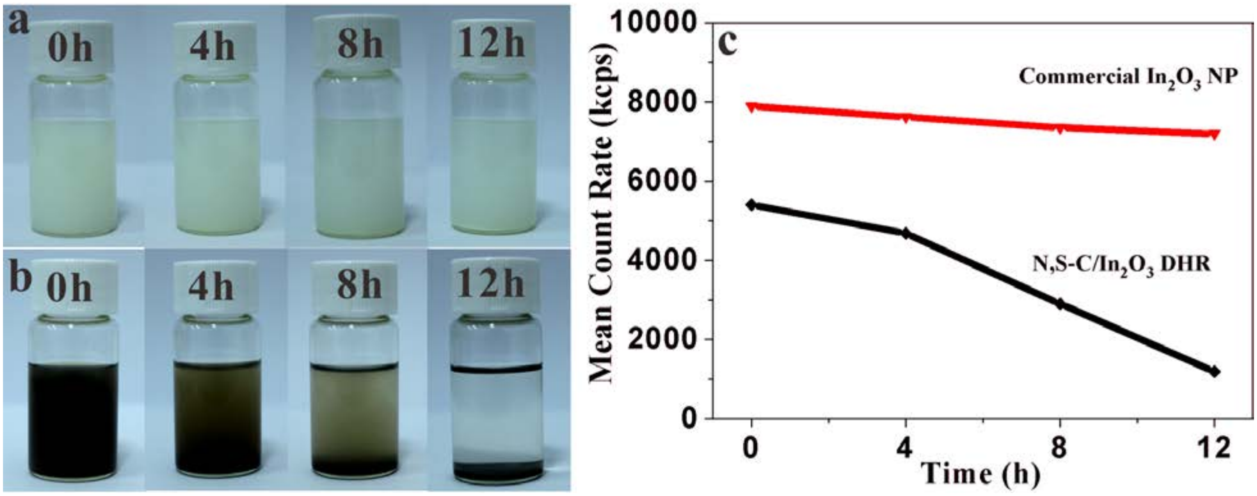

Supplementary Figure 17. Sedimentation tests. Sedimentation of (a) commercial In_2O_3 NP and (b) N,S-C/In_2O_3 DHR for different times without agitation, and (c) plots of mean count rates for different sedimentation times without agitation.

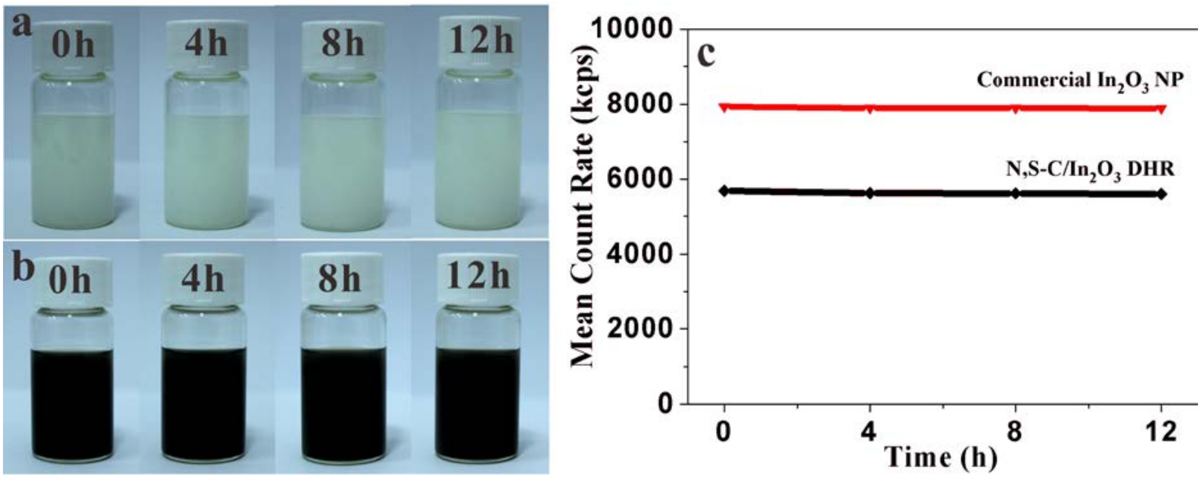

Supplementary Figure 18. Sedimentation tests. Dispersity of (a) commercial In_2O_3 NP and (b) N,S-C/In_2O_3 DHR for different times with stirring, and (c) plots of mean count rates for different sedimentation times with stirring.

The quantum yield is also an important index for evaluating photocatalyst activity. The catalyst solution was irradiated under blue LED (λ = 450 nm, 3W) for 12 h. The average light density was ca. 2.35 mW·cm^{-2} and the irradiation area was 4.27 cm^2. In the photocatalytic process, 0.1 mmol phenylboronic acid was used and completely converted into phenol after 12 h. Therefore, the quantum yield calculated was 0.0614.

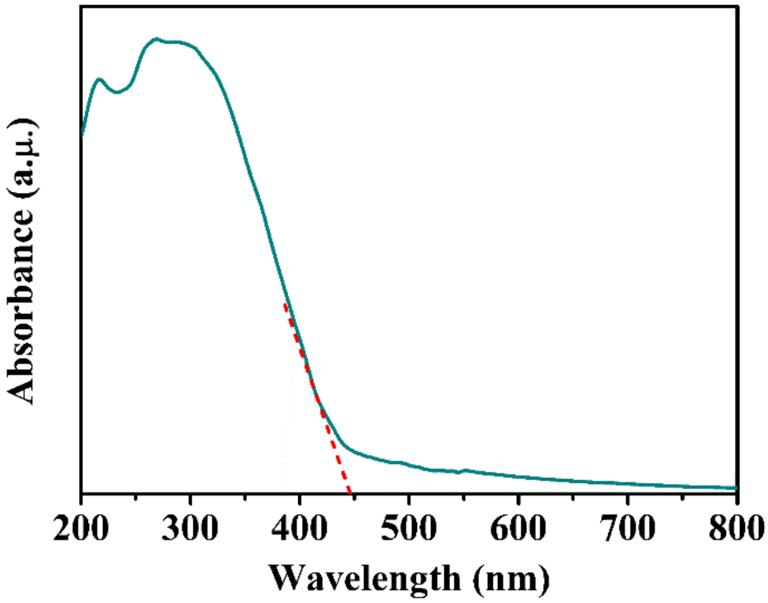

Supplementary Figure 19. UV-vis absorption spectrum of In_2O_3 DHR. Source data are provided as a Source Data file.

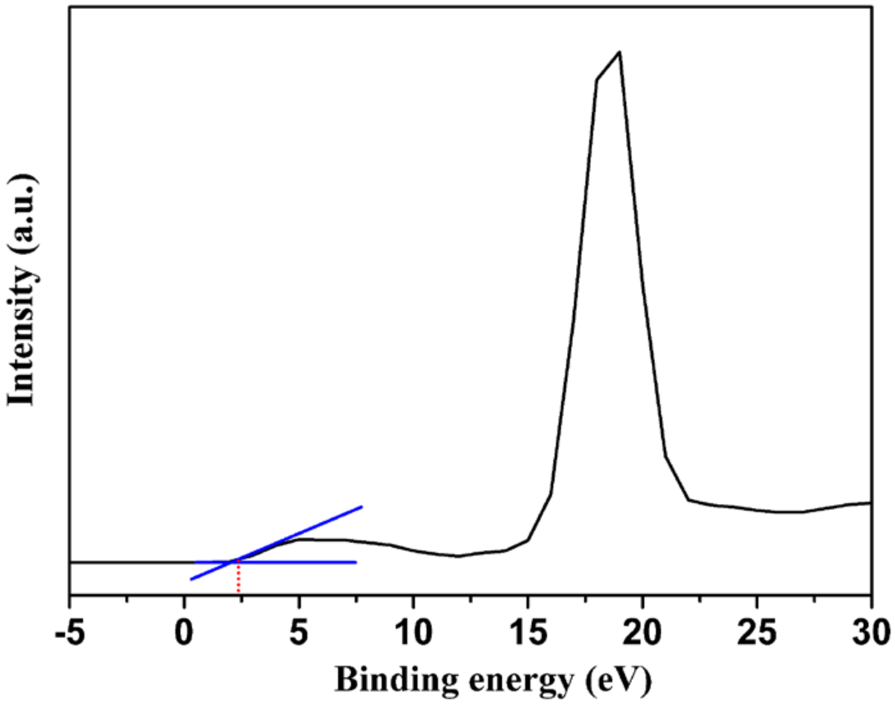

Supplementary Figure 20. VB XPS spectrum of pure In_2O_3. Source data are provided as a Source Data file.

The valence band X-ray photoelectron spectroscopy (VB XPS) of pure In_2O_3 shows that the position of the VB edge of In_2O_3 is located at about 2.15 eV, which is consistent with the value calculated from the Mulliken electronegativity theory.

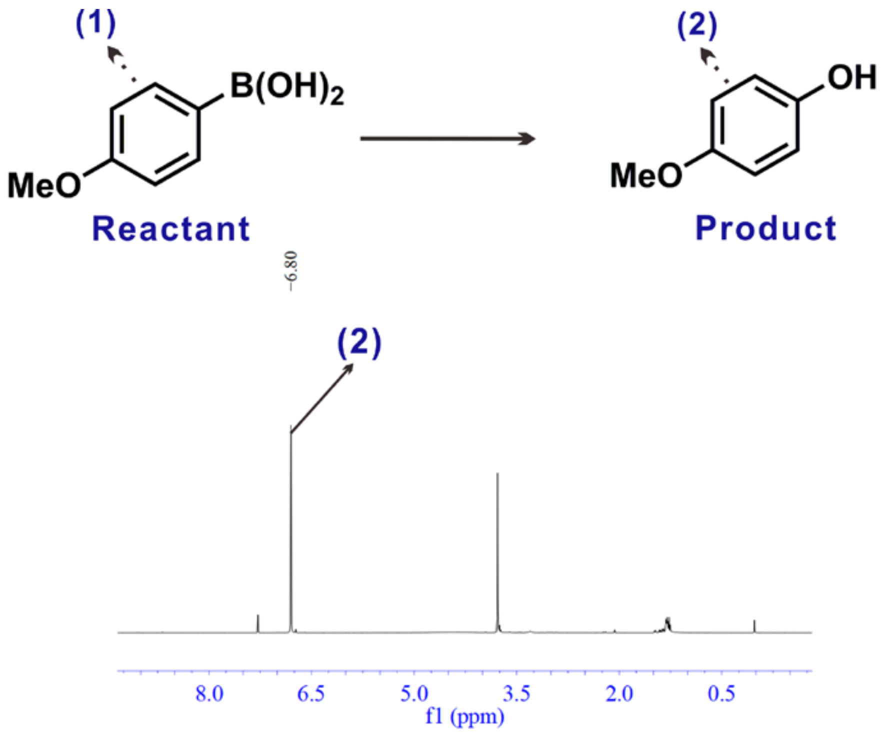

Supplementary Figure 21. ^{1}H NMR spectrum of oxidative hydroxylation of arylboronic acid without a scavenger. Source data are provided as a Source Data file.

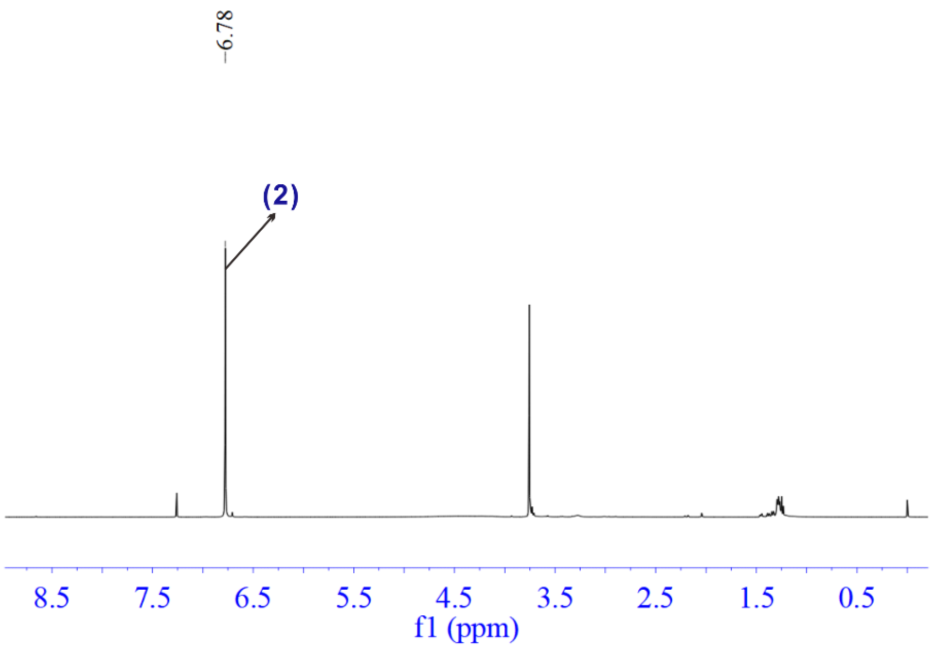

Supplementary Figure 22. ^{1}H NMR spectrum of oxidative hydroxylation of arylboronic acid with T-BuOH. Source data are provided as a Source Data file.

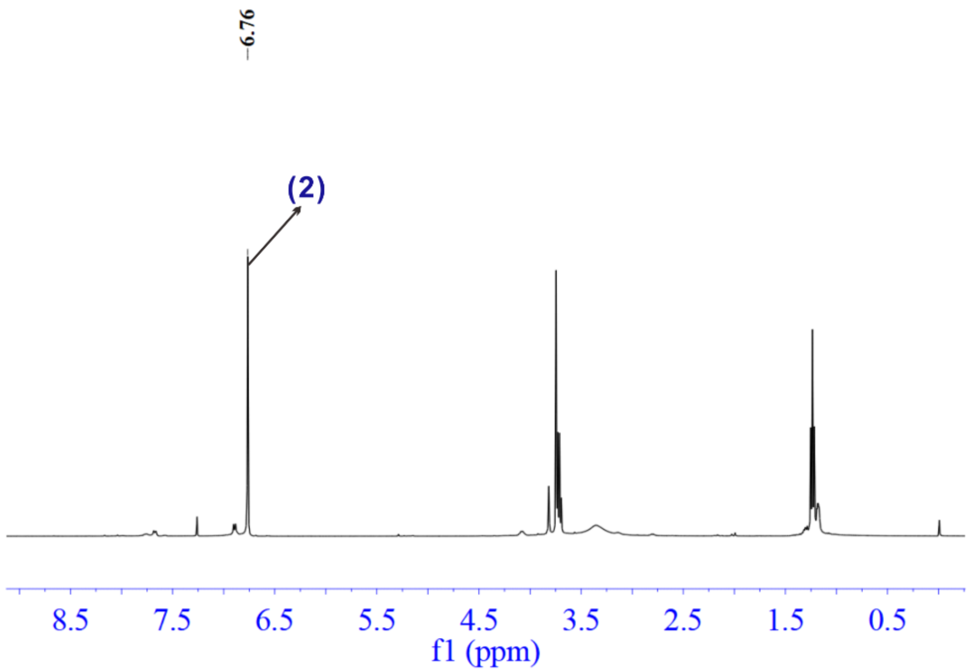

Supplementary Figure 23. ^{1}H NMR spectrum of oxidative hydroxylation of arylboronic acid with TEA. Source data are provided as a Source Data file.

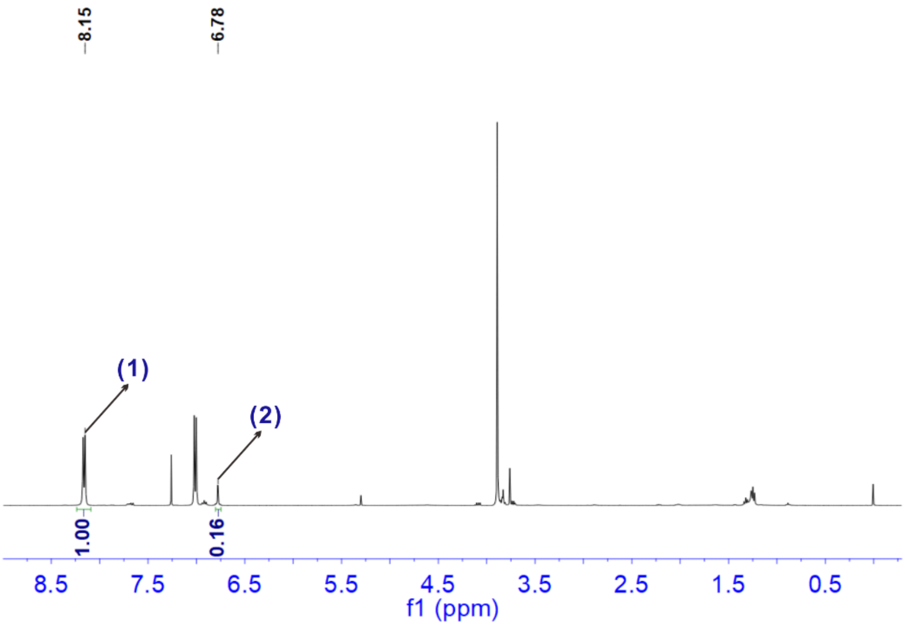

Supplementary Figure 24. ^{1}H NMR spectrum of oxidative hydroxylation of arylboronic acid with TEMPO. Source data are provided as a Source Data file.

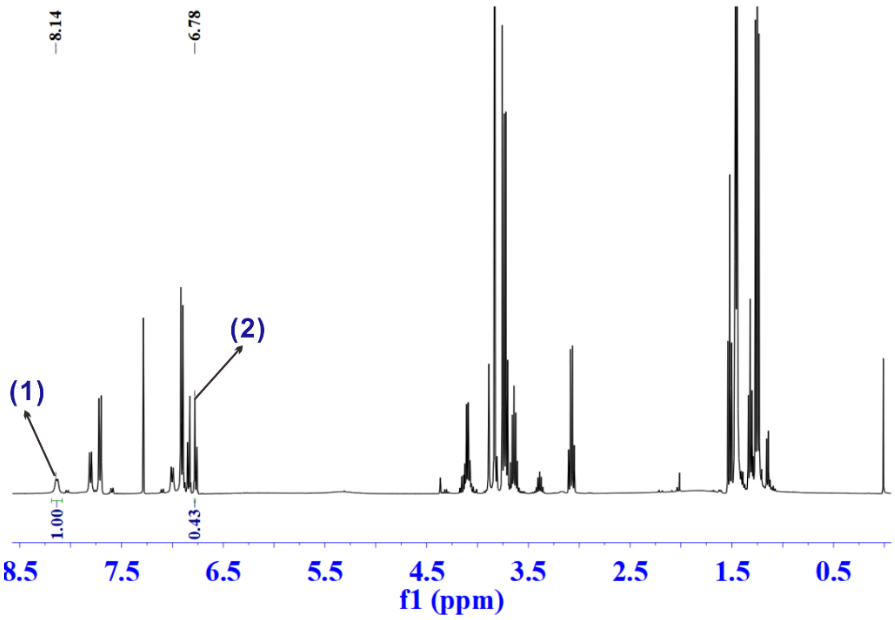

Supplementary Figure 25. ^{1}H NMR spectrum of oxidative hydroxylation of arylboronic acid with CCl_4. Source data are provided as a Source Data file.

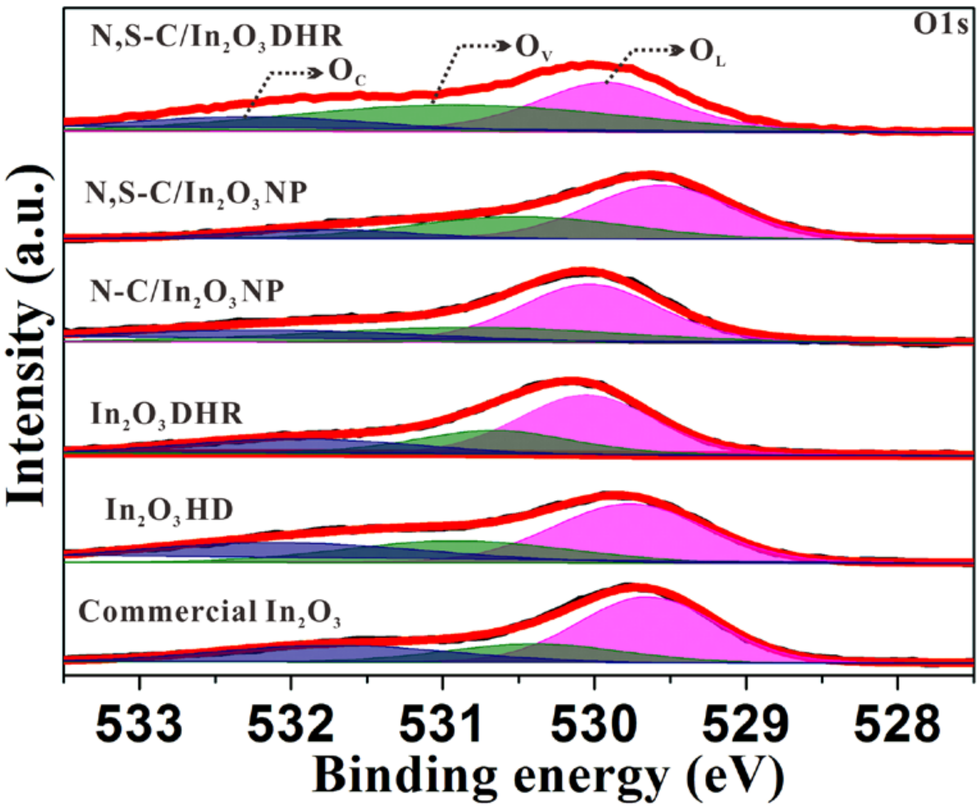

Supplementary Figure 26. XPS spectra and curve fitting of O 1s in different samples indicated.

Supplementary Tables

Supplementary Table 1. Photocatalyzed oxidative hydroxylation of arylboronic acids in different reaction conditions. Source data are provided as a Source Data file.

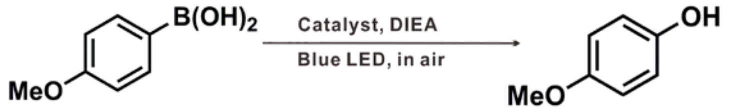

hυ (450nm, LED)	Catalyst (DNHR)	DIEA	Reaction time	Conversion (%)
No	Yes	Yes	12 h	3
Yes	No	Yes	12 h	7
Yes	Yes	No	12 h	0

The comparative experiments were performed in the absence of light, DIEA and catalyst to determine the conditions for this photocatalytic reaction. The result was shown in Table S1, indicating that only a trace of the product was obtained without any of them (light, DIEA and catalyst). Therefore, this photocatalytic reaction was carried out under blue LED irradiation (λ = 450 nm, 3 W) using DIEA as a co-catalyst in a Schlenk tube.

Supplementary Table 2. Curve fitting results of O 1s spectra in six different In_2O_3 samples.

Samples	O_L (In-O)	O_V (Vacancy)	O_C (Chemisorbed)
Commercial In_2O_3	529.6<br>55.5 %	530.4<br>20.6 %	531.7<br>23.9 %
In_2O_3 HD	529.6<br>46.5 %	530.6<br>24.4 %	531.8<br>29.1 %
In_2O_3 DHR	529.9<br>52.7 %	530.6<br>26.8 %	531.8<br>20.5 %
N-C/In_2O_3 NP	529.8<br>49.7%	530.7<br>29.2%	531.9<br>21.1%
N,S-C/In_2O_3 NP	529.6<br>56.4%	530.6<br>32.4%	531.9<br>11.2%
N,S-C/In_2O_3 DHR	529.7<br>39.2%	530.7<br>41.9%	531.9<br>18.8%
